# Catalytic trifluoromethylation of iodoarenes by use of 2-trifluoromethylated benzimidazoline as trifluoromethylating reagent

**DOI:** 10.3762/bjoc.16.198

**Published:** 2020-09-30

**Authors:** Tatsuhiro Uchikura, Nanami Kamiyama, Taisuke Ishikawa, Takahiko Akiyama

**Affiliations:** 1 Department of Chemistry, Faculty of Science, Gakushuin University, Mejiro, Toshima-ku, Tokyo, 171-8588, Japan

**Keywords:** benzimidazoline, catalysis, copper, fluorine chemistry, trifluoromethylation

## Abstract

The trifluoromethylation of iodoarenes was accomplished by use of a 2-trifluoromethylbenzimidazoline derivative as the trifluoromethylating reagent and a catalytic amount of Cu(I) in the presence of 2,2'-bipyridyl as the ligand. Through a mechanistic study, we found that the oxidative addition of the iodoarene to the Cu(I)–CF_3_ species is the rate-determining step.

## Introduction

**Figure 1:**
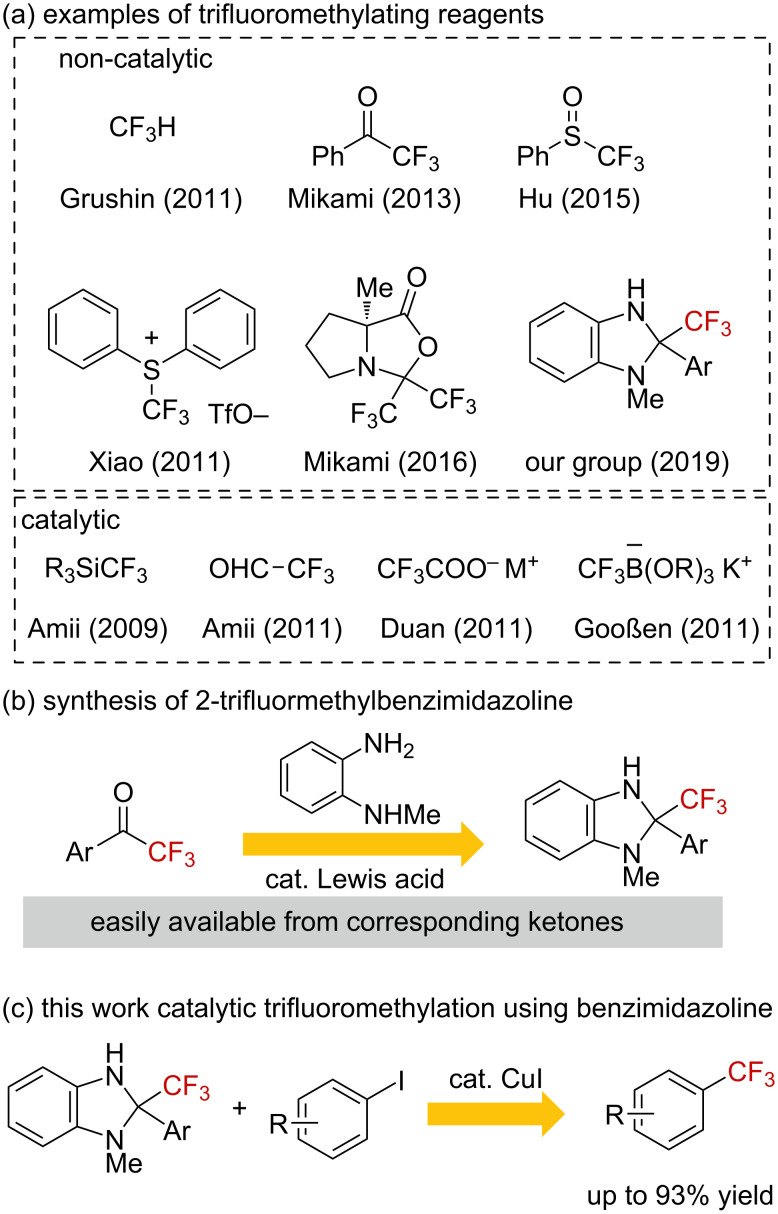
Trifluoromethylation of aryl halides.

The introduction of a trifluoromethyl group is one of the most attractive reactions in drug discovery [1–2]. In the past decade, trifluoromethylation reactions of aryl halides in the presence of transition-metal complexes were reported [3–21]. CuCF_3_ is a useful species for the trifluoromethylation of aryl halides and there are a number of precursors of CuCF_3_ for trifluoromethylation reactions. In contrast, the catalytic generation of CuCF_3_ was less investigated [15–21]. For example, R_3_SiCF_3_, a fluoral derivative, and trifluoroacetates were employed as precursors of CuCF_3_ species for the catalytic trifluoromethylation of iodoarenes (Figure 1a) and the development of novel types of trifluoromethylating reagents is still desired. We have recently reported the trifluoromethylation of iodoarenes by use of 2-aryl-2-trifluoromethylbenzimidazoline as the trifluoromethylating reagent in the presence of 3 equiv of a copper salt [22]. The benzimidazoline derivatives could be readily prepared from relatively cheap materials, namely, trifluoromethylacetophenone and phenylenediamine derivatives (Figure 1b). Herein we report a catalytic trifluoromethylation of iodoarenes by use of benzimidazoline derivatives in the presence of a catalytic amount of copper salts and a bipyridyl ligand (Figure 1c).

## Results and Discussion

We first investigated the reaction conditions by use of *p*-iodonitrobenzene (**1a**) and 2-phenyl-2-trifluoromethyl-1-methylbenzimidazoline (**2**) (Table 1). Using 3 equiv of CuI gave 4-trifluoromethyl-1-nitrobenzene (**3a**) quantitatively, as we had reported previously (Table 1, entry 1) [22]. Decreasing the CuI catalyst loading to 20 mol % lowered the yield of **3a** even at a high temperature (Table 1, entries 2 and 3). In order to stabilize the copper(I) catalyst, a bipyridyl ligand was employed in the reaction [23]. By this, the trifluoromethylation proceeded with a catalytic amount of CuI in the presence of 0.8 equiv of 2,2’-bipyridyl to furnish product **3a** in 80% yield (Table 1, entry 4). However, smaller amounts of CuI (10 mol %) and 2,2’-bipyridyl (20 mol %) gave compound **3a** in lower yields (Table 1, entries 5 and 6). Benzonitrile was the solvent of choice (Table 1, entries 7 and 8). Replacing CuI by CuBr or CuCl gave similar results (Table 1, entries 9 and 10). However, we selected the most stable and readily available CuI as the catalyst for further investigations. Thus, the trifluoromethylation of *p*-iodonitrobenzene proceeded by use of 2-phenyl-2-trifluoromethyl-1-methylbenzimidazoline in the presence of catalytic amounts of CuI and 2,2’-bipyridyl as ligand.

**Table 1:** Screening for reaction conditions^a^.

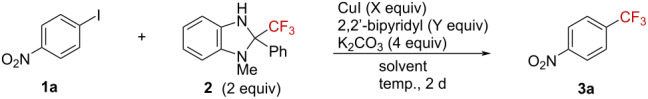					

entry	X	Y	solvent	temp.	yield^b^

1	3	0	PhCN	60 °C	quant
2	0.2	0	PhCN	60 °C	36%
3	0.2	0	PhCN	90 °C	40%
4	0.2	0.8	PhCN	90 °C	80%
5	0.1	0.8	PhCN	90 °C	59%
6	0.2	0.2	PhCN	90 °C	63%
7	0.2	0.8	MeCN	90 °C	32%
8	0.2	0.8	DMF	90 °C	30%
9^c^	0.2	0.8	PhCN	90 °C	72%
10^d^	0.2	0.8	PhCN	90 °C	79%

^a^Performed with **2a** (0.050 mmol) and **1a** (0.10 mmol) in solvent (1.0 mL). ^b^Determined by ^19^F NMR spectroscopy (benzotrifluoride was used as the internal standard). ^c^CuBr was used instead of CuI. ^d^CuCl was used instead of CuI.

We next screened for 2,2’-bipyridyl ligands to be used with CuI (Table 2). 4,4’-Dimethyl-2,2’-bipyridyl (**4b**) gave product **3a** in a higher yield than 2,2’-bipyridyl (**4a**), whereas 5,5’- and 6,6’-dimethylbipyridyl (**4c** and **4d**) were not effective. From these results, the substituents at 4,4’-positions were expected to be beneficial for the reaction, and other 4,4’-substituted bipyridyl ligands were investigated. We found that 4,4’-di-*tert*-butyl-2,2’-bipyridyl (**4e**) afforded the best result (91% yield). On the other hand, 2,2'-bipyridyl ligands bearing electron-donating (–OMe, **4f**) and -withdrawing (–CF_3_, **4g**) groups furnished **3a** in low yields. Moreover, other electron-withdrawing ligands, i.e., phenanthroline derivatives (**4h** and **4i**), and electron-donating ligands such as 2,2’-biimidazole (**4j**) and tetramethylethylenediamine (**4k**) gave inferior results.

**Table 2:** Screening for diamine ligands^a^.

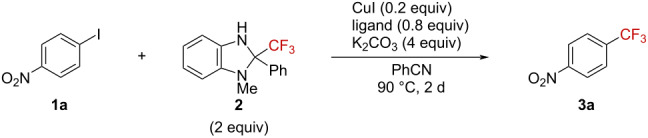			

ligand	yield^b^	ligand	yield^b^

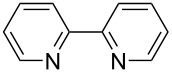 **4a**	80%	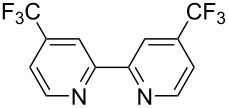 **4g**	56%
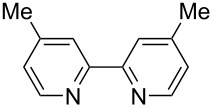 **4b**	82%	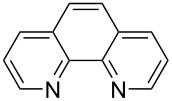 **4h**	64%
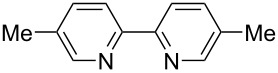 **4c**	72%	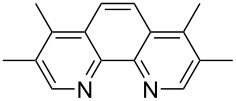 **4i**	57%
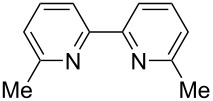 **4d**	19%	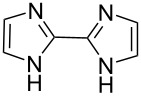 **4j**	trace
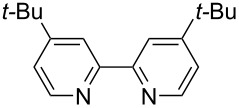 **4e**	91%	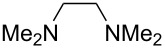 **4k**	26%
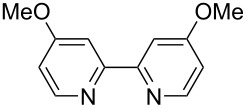 **4f**	73%	–	40%

^a^Performed with **2a** (0.050 mmol) and **1a** (0.10 mmol) in solvent (1.0 mL). ^b^Determined by ^19^F NMR spectroscopy (benzotrifluoride was used as the internal standard).

**Figure 2:**
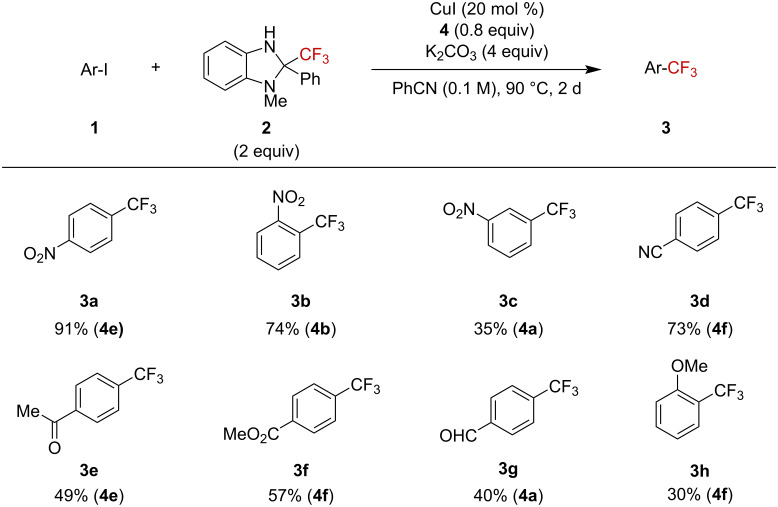
Scope of aryl iodides. Yields determined by ^19^F NMR spectroscopy and used ligand is given in parenthesis.

We next screened for the generality of the reaction towards various substrates under the optimized conditions (Figure 2). Electron-deficient aryl iodides were well tolerated furnishing the corresponding trifluoromethylation products in high yields. Among the tested nitrophenyl derivatives, *p*- and *o-*nitrophenyliodide gave the products in highest yields. In contrast, *m*-iodonitrobenzene afforded the trifluoromethylated product in a decreased yield of 35% due to the higher electron density of the *meta*-position compared to the *ortho-* and *para*-positions. Iodoarenes bearing other electron-withdrawing substituents, such as *p-*cyano, *p-*acetyl, and *p-*methoxycarbonyl, were also suitable and gave products **3d**–**f** in moderate to high yields. Furthermore, the presence of a formyl group was also tolerated in the reaction, and *p-*formyltrifluoromethylbenzene (**3g**) was obtained in 40% yield. However, the electron-rich substrate 2-methoxyiodobenzene (**1h**) gave product **3h** in only a modest yield (30%).

**Figure 3:**
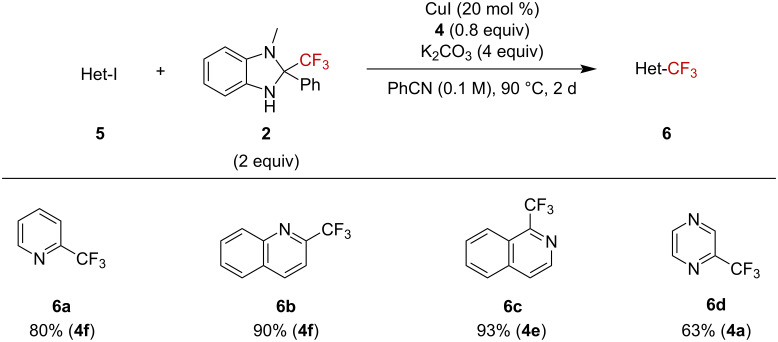
Scope of heteroaryl iodides. Yields determined by ^19^F NMR spectroscopy and used ligand is given in parenthesis.

Heteroaryl iodides were also suitable substrates (Figure 3). 2-Iodopyridine (**5a**) gave the expected trifluoromethylation product **6a** in 80% yield. 2-Iodoquinoline (**5b**) and 1-iodoisoquinoline (**5c**) were also suitable substrates to furnish desired products **6b** and **6c** in high yields. Furthermore, iodopyrazine was applicable to furnish **6d** in 63% yield.

Finally, a mechanistic study of the reaction was carried out. First, the active species of the reaction was investigated by NMR analysis. The generation of Cu(I)–CF_3_ was observed by mixing benzimidazoline **2** and CuI in EtCN at 90 °C (Figure S1, in Supporting Information File 1). Therefore, CuI and **2** generated CuCF_3_ species as the active species for the trifluoromethylation [24].

**Figure 4:**
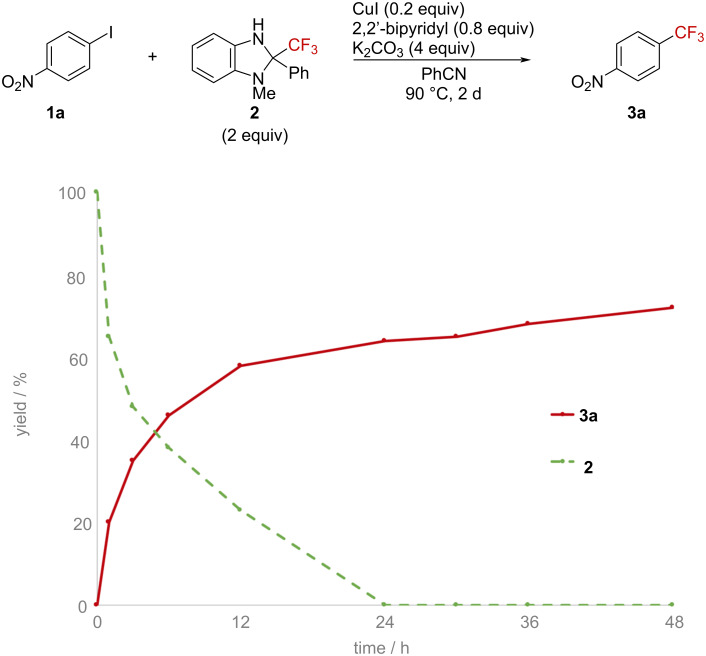
Time course of the trifluoromethylation reaction.

Then, the dependence of the conversion on the reaction time was estimated (Figure 4) and no induction period was observed. Although benzimidazoline **2** was completely consumed after 24 h, the yield of the trifluoromethylation product continued to increase up to 48 h. This suggests that product generation proceeded slower than the cleavage of the C–CF_3_ bond of benzimidazoline.

**Figure 5:**
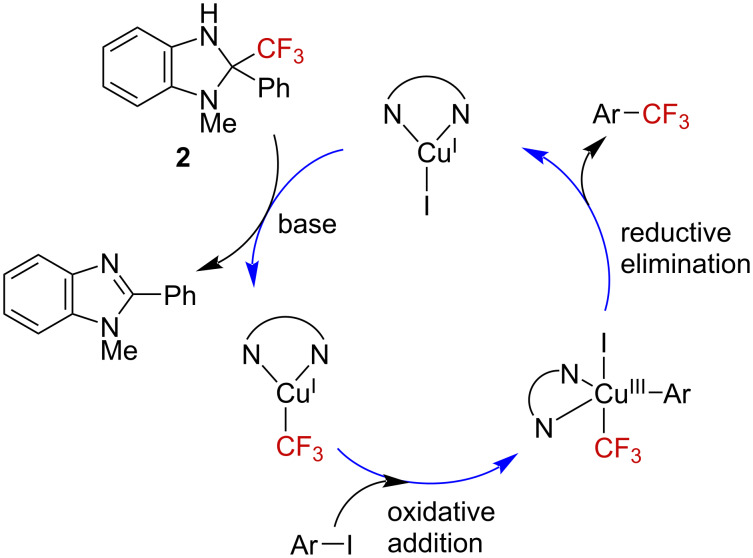
Proposed mechanism of the catalytic cycle.

We propose a mechanism for the reaction, as shown in Figure 5. The Cu(I)–CF_3_ species, generated through the reaction of benzimidazoline **2** with CuI under basic conditions, underwent an oxidative addition reaction with the aryl iodide to generate a Cu(III) complex. A subsequent reductive elimination furnished the trifluoromethylarene and Cu(I). Because an electron-donating ligand was more effective than an electron-deficient one (Table 2), and the reaction with benzimidazoline proceeded rapidly (Figure 4), the oxidative addition was suggested to be the rate-determining step.

## Conclusion

In conclusion, we have developed a catalytic trifluoromethylation of aryl iodides by using trifluoromethylated benzimidazoline derivatives. The mechanistic study revealed that the oxidative addition was the rate-determining step of this reaction. 2-Phenyl-2-trifluoromethyl-1-methylbenzimidazoline is a novel type of trifluoromethylating reagents that might be useful for organic synthesis.

## Experimental

General procedure of trifluoromethylation: Aryl iodide **1** (0.1 mmol), **2** (56 mg, 0.2 mmol), CuI (3.8 mg, 0.02 mmol), 2,2’-bipyridyl (12.5 mg, 0.08 mmol), and potassium carbonate (55.6 mg, 0.4 mmol) were mixed in benzonitrile (1.0 mL), and the mixture heated to 90 °C. After 48 h, hexafluorobenzene was added as an internal standard and the mixture analyzed by ^19^F NMR spectroscopy for the calculation of the NMR yield. Then, the crude products were purified by preparative TLC to give pure products **3**.

## Supporting Information

File 1 Details of screening experiments, synthetic procedures and characterization data of new compounds, and copies of spectra.
