# Supplementary material for: Catalytic trifluoromethylation of iodoarenes by use of 2-trifluoromethylated benzimidazoline as trifluoromethylating reagent
Source: Beilstein J Org Chem. 2020 Sep 30;16:2442–7. doi: 10.3762/bjoc.16.198 (PMC7537379; doi:10.3762/bjoc.16.198)
Supplement: File 1 — Details of screening experiments, synthetic procedures and characterization data of new compounds, and copies of spectra. [file Beilstein_J_Org_Chem-16-2442-s001.pdf]

BEILSTEIN JOURNAL OF ORGANIC CHEMISTRY

Supporting Information

for

Catalytic trifluoromethylation of iodoarenes by use of 2-trifluoromethylated benzimidazoline as trifluoromethylating reagent

Tatsuhiro Uchikura, Nanami Kamiyama, Taisuke Ishikawa and Takahiko Akiyama

Beilstein J. Org. Chem. **2020,** *16,* 2442–2447. doi:10.3762/bjoc.16.198

Details of screening experiments, synthetic procedures and characterization data of new compounds, and copies of spectra

License and Terms: This is a supporting information file under the terms of the Creative Commons Attribution License (https://creativecommons.org/licenses/by/4.0). Please note that the reuse, redistribution and reproduction in particular requires that the authors and source are credited. The license is subject to the *Beilstein Journal of Organic Chemistry* terms and conditions: (https://www.beilstein-journals.org/bjoc)

Table of contents	Page
1. General methods	S2
2. Additional data	
2-1. Screening of detailed conditions of trifluoromethylation	S3
2-2. NMR study	S8
2-3. Time profile of trifluoromethylation	S9
3. Synthetic procedures and characterization of new compounds	
3-1. Synthesis of trifluoromethylated benzimidazoline derivatives	S10
3-2. Trifluoromethylation of iodoarenes	S12
4. References	S17
5. Spectral data	S18

1. General methods

All operations were performed under air unless otherwise noted. NMR spectra of products (^{1}H and ^{13}C) were recorded on a Bruker AVANCE-III (400 MHz for ^{1}H, 100 MHz for ^{13}C, 376 MHz for ^{19}F) and a JEOL ECZ-400 (400 MHz for ^{1}H, 100 MHz for ^{13}C, 376 MHz for ^{19}F) spectrometer using $CDCl_3$ [tetramethylsilane (0 ppm) served as an internal standard in ^{1}H NMR and $CDCl_3$ (77.0 ppm) in ^{13}C NMR, hexafluorobenzene (−63.9 ppm) or benzotrifluoride (−63.7 ppm) served as an internal or external standard in ^{19}F NMR]. Chemical shifts are expressed in parts per million (ppm). IR spectra were recorded on an FT/IR-4200 (JASCO Co., Ltd.). ESI mass analyses were performed on Bruker micrOTOF mass spectrometer.

All solvents were distilled according to the usual procedures and stored over molecular sieves unless otherwise noted. All substrates were purified by distillation (for liquids) or recrystallization (for solids). Benzimidazoline derivative **2**[S1] and aryl iodides (**5b**, **5c**)[S2, S3] were synthesized according to the literature procedures. Other chemicals were purchased and used as received.

2. Additional data

2-1. Screening of detailed conditions of trifluoromethylation

Table S1: Screening of the loading of copper (I) salt.

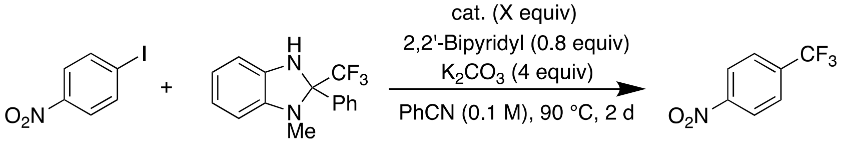

1a **2** (2 equiv) **3a**

entry	cat.	X	yield
1	CuI	1	65%
2	CuI	0.5	70%
3	CuI	0.3	76%
4	CuI	0.2	80%
5	CuI	0.1	59%
6	CuI	0.05	65%
7	CuCl	0.2	79%
8	CuBr	0.2	72%
9	$(CuI)_4$•3(Me_2S)	0.2	63%

Reactions were carried out according to Procedure I on Page S12. Twenty mol % of copper(I) iodide gave the best result (80% yield) according to the screening of the loading of copper(I) iodide. Further lowering the loading of the catalyst decreased the yields of the trifluoromethylated product.

Copper (I) iodide was the best catalyst for the promotion of the trifluoromethylation reaction (entries 4, 7–9).

• Equivalent of ligand.

Table S2: Screening of loading of the ligand **4**.

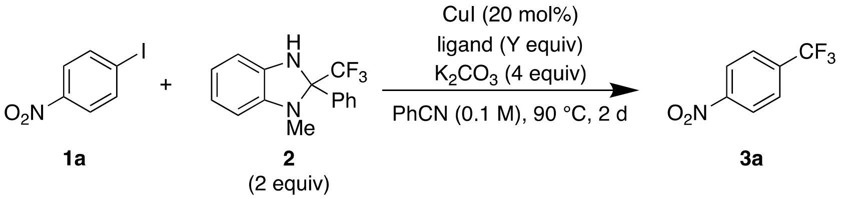

entry	ligand	Y	yield
1	**4a**	0.2	63%
2	**4a**	0.4	71%
3	**4a**	0.8	80%
4	**4a**	1	71%
5	**4e**	0.2	76%
6	**4e**	0.4	82%
7	**4e**	0.8	91%
8	**4e**	1	78%

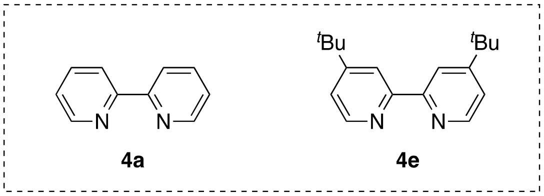

Reactions were carried out according to Procedure I on Page S12. The most favorable amount of ligand was 4 equiv of CuI (entries 3 and 7).

- Screening of base

Table S3: Screening of base.

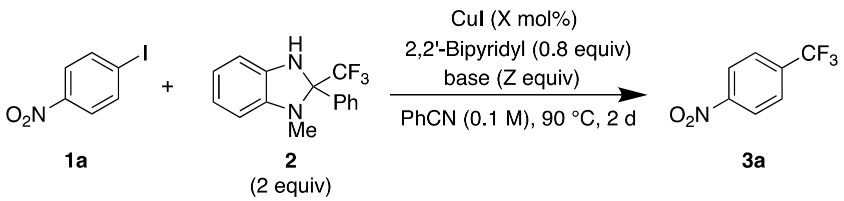

entry	X	base	Z	yield
1	10	K_2CO_3	4	59%
2	10	Cs_2CO_3	4	29%
3	10	Na_2CO_3	4	6%
4	20	K_2CO_3	2	66%
5	20	K_2CO_3	4	80%
6	20	K_2CO_3	6	75%

Reactions were carried out according to Procedure I on Page S12. Potassium carbonate was the most suitable base for the trifluoromethylation (entries 1,4–6).

- Screening of solvent

Table S4: Screening of solvent.

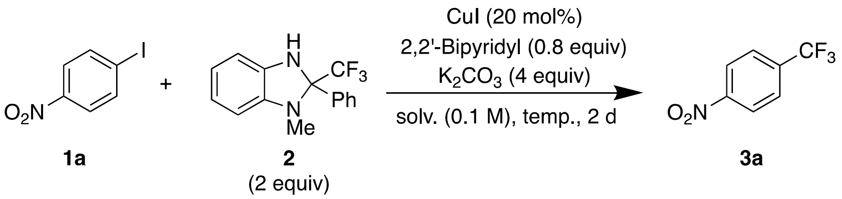

entry	solv.	temp.	yield
1	DMF	90 °C	30%
2	MeCN	90 °C	32%
3	EtCN	90 °C	51%
4	PhCN	90 °C	80%
5	PhCN	60 °C	36%
6	PhCN	80 °C	60%
7	PhCN	100 °C	70%

Reactions were carried out according to Procedure I on Page S12. According to the screening of polar solvents, benzonitrile gave the best result and the trifluoromethylated product **3a** was obtained in 80% yield. Other solvents afforded lower yields.

- Screening of the aryl substituents at 2-position of benzimidazoline.

Table S5: Screening of aryl groups at the 2-position of benzimidazoline **2**.

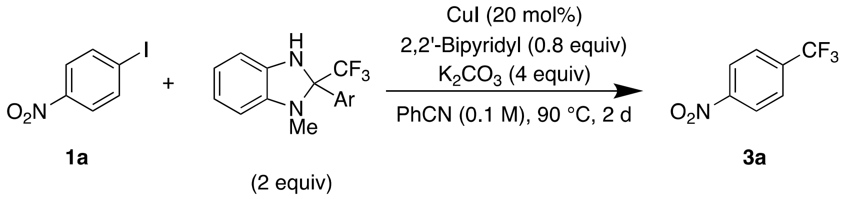

entry	Ar	yield
1	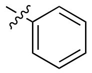	80%
2	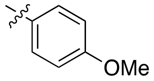	66%
3	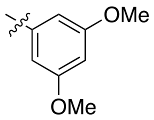	74%
4	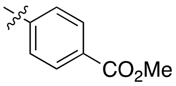	57%
5	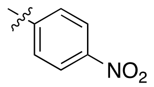	37%

Reactions were carried out according to Procedure I on Page S12. Using benzimidazolidines bearing an electron-donating aryl group, the yield of the product remained almost unchanged compared with a phenyl group (entries 1–3).

In contrast, the presence of an electron-deficient group decreased the reactivity because the nucleophilicity of CF_3 group was decreased by the electron-deficient aryl group (entries 4 and 5). This means the oxidative addition step proceeds by nucleophilic process.

2-2. NMR study

Procedure: In an NMR sample tube, **2** (14.0 mg, 0.05 mmol), CuI (9.5 mg, 0.05 mmol), and potassium carbonate (13.9 mg, 0.10 mmol) were mixed in propionitrile (0.75 mL), and the mixture was warmed at 60 °C. After 2 h, a ^{19}F NMR spectrum was recorded and the formation of $CuCF_3$ was observed due to the appearance of the signal at –27.6 ppm.[S4] According to the results, $CuCF_3$ is suggested to be a key intermediate of the trifluoromethylation reaction.

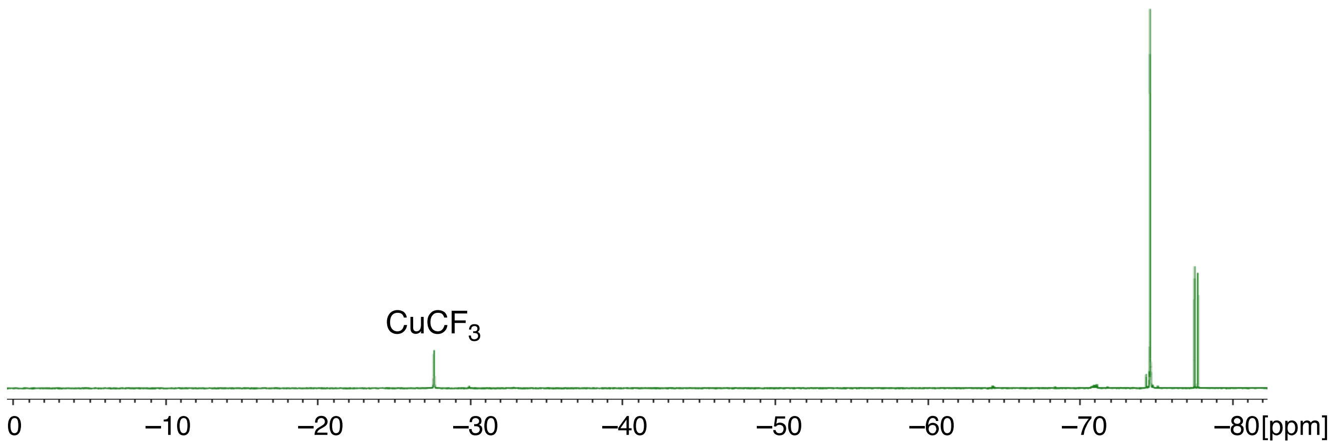

Figure S1: ^{19}F NMR spectrum for observation of reactive intermediate (reference was set by hexafluorobenzene as external standard).

2-3. Time profile of trifluoromethylation

Table S6: Dependence on the reaction time.

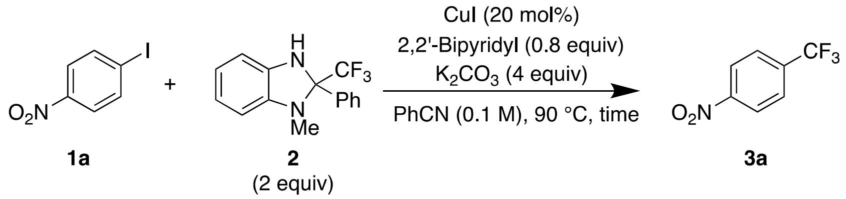

entry	time	yield	imidazoline recovery
1	1 h	20%	65%
2	3 h	35%	48%
3	6 h	59%	18%
4	24 h	64%	0%
5	30 h	65%	0%
6	48 h	80%	0%

Reactions were carried out according to Procedure I on Page S12. The time profile chart is shown in Figure 4 in the main text. Although benzimidazoline **2** was completely consumed after 24 h reaction time, the yield of trifluoromethylation product increased up to 48 h. Therefore, the generation of the product was suggested to be slower than the cleavage of C–CF_3 bond of benzimidazoline.

3. Synthetic procedures and characterization of new compounds

3-1. Synthesis of trifluoromethylated benzimidazoline derivatives

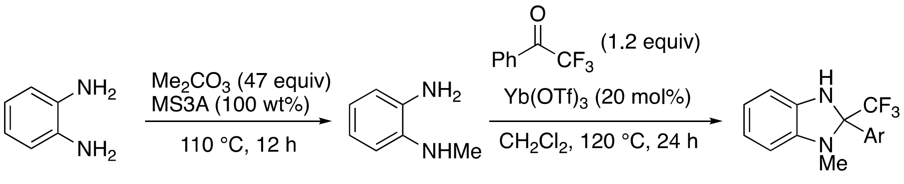

<u>**Synthesis of trifluoromethylated benzimidazolines**</u>

***N*-Methyl-1,2-phenylenediamine (S1)**[S1)] Under a nitrogen atmosphere, a mixture of 1,2-phenylenediamine (28 mmol), dimethyl carbonate (47 equiv), and MS 3Å (100 wt %) was heated at 110 °C for 12 h, and the reaction was monitored by TLC. After completion of the reaction, the supernatant of the reaction mixture was purified by column chromatography (hexane/AcOEt 2:1) to give **S1** (77%).

***N*-Methyl-2-phenyl-2-trifluoromethylbenzimidazoline, 2 and S2**[S1)] Under a nitrogen atmosphere, a mixture of **S1** (8.2 mmol), 2,2,2-trifluoroacetophenone (1.2 equiv), $Yb(OTf)_3$ (20 mol %), MS 3Å (100 wt %) in dichloromethane (9.0 mL) at 120 °C under autoclave conditions. After 24 h, the reaction was quenched by filtration through a Celite pad and washing with AcOEt. The solution was evaporated in vacuo, and a mixture of products and 2,2,2-trifluoroacetophenone was isolated by column chromatography (hexane/AcOEt 10:1), followed by recrystallization from hexane.

Data of products

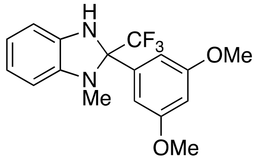

S2b

^{1}H NMR (400 MHz, $CDCl_3$) δ 2.78 (s, 3H), 3.78 (s, 6H), 4.23 (br, 1H), 6.37 (d, J = 7.6 Hz, 1H), 6.48 (t, J = 2.4 Hz, 1H), 6.57 (d, J = 6.8 Hz, 1H), 6.62 (t, J = 7.2 Hz, 1H), 6.71 (s, 2H), 6.79 (t, J = 7.6 Hz, 1H) ppm.

^{13}C NMR (100 MHz, $CDCl_3$) δ 30.4, 55.4, 86.3 (q, J = 28.8 Hz), 100.6, 104.3, 105.0 (d, J = 2.0 Hz), 108.4, 118.3, 121.1, 124.5 (q, J = 290 Hz), 136.7, 138.2, 141.0, 161.1 ppm.

^{19}F NMR (376 MHz, $CDCl_3$, external standard: C_6F_6; −163.9 ppm) δ −74.3 (s, 3F) ppm.

IR (KBr, cm^{-1}) 2359, 2342, 1602, 1502, 1208, 1158.

LRMS: *m/z* = 337 $[M–H]^+$, HRMS (ESI) Calcd for $C_{17}H_{16}F_3N_2O_2$: 337.1158. Found 337.1163.

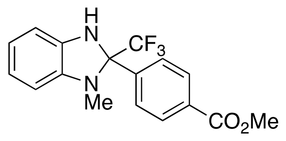

S2c

^{1}H NMR (400 MHz, $CDCl_3$) δ 2.73 (s, 3H), 3.94 (s, 3H), 4.31 (brs, 1H), 6.40 (d, *J* = 7.2 Hz, 1H), 6.61 (d, *J* = 7.2 Hz, 1H), 6.65 (t, *J* = 7.6 Hz, 1H), 6.81 (t, *J* = 7.6 Hz, 1H), 7.66 (d, *J* = 8.0 Hz, 2H), 8.07 (d, *J* = 8.4 Hz, 2H) ppm.

^{13}C NMR (100 MHz, $CDCl_3$) δ 30.389, 30.399, 52.4, 86.1 (q, *J* = 29.6 Hz), 104.5, 108.2, 118.6, 121.2, 124.4 (q, *J* = 290 Hz), 126.79, 126.81, 130.1, 131.2, 136.6, 140.57, 140.64, 166.3 ppm.

^{19}F NMR (376 MHz, $CDCl_3$, external standard: C_6F_6; −163.9 ppm) δ −74.4 (s, 3F) ppm.

IR (KBr, cm^{-1}) 3354, 1726, 1502, 1286, 1242, 1200, 1170, 1114, 1018, 936, 736.

LRMS (ESI): *m/z* = 335 $[M–H]^+$, HRMS (ESI) Calcd for $C_{17}H_{14}F_3N_2O_2$: 335.1002. Found 335.1010.

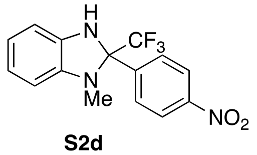

S2d

^{1}H NMR (400 MHz, $CDCl_3$) δ 2.76 (s, 3H), 4.38 (brs, 1H), 6.42 (d, *J* = 7.6 Hz, 1H), 6.60 (d, *J* = 7.2 Hz, 1H), 6.68 (t, *J* = 7.2 Hz, 1H), 6.82 (t, *J* = 7.2 Hz, 1H), 7.80 (d, *J* = 8.4 Hz, 2H), 8.27 (d, *J* = 8.8 Hz, 2H) ppm.

^{13}C NMR (100 MHz, $CDCl_3$) δ 30.2, 85.8 (q, *J* = 29.6 Hz), 104.7,108.2, 119.0, 121.3, 124.0, 124.3 (q, *J* = 291 Hz), 128.0 (d, *J* = 2.4 Hz), 136.1, 140.1, 142.7, 148.4 ppm.

^{19}F NMR (376 MHz, $CDCl_3$, external standard: C_6F_6; −163.9 ppm) δ −74.1 (s, 3F) ppm.

IR (KBr, cm^{-1}) 2360, 1523, 1500, 1352, 1170.

LRMS (ESI): *m/z* = 322 $[M–H]^+$, HRMS (ESI) Calcd for $C_{15}H_{11}F_3N_3O_2$: 322.0798. Found 322.0797.

3-2. Trifluoromethylation of iodoarenes

General procedure of trifluoromethylation (Procedure I)

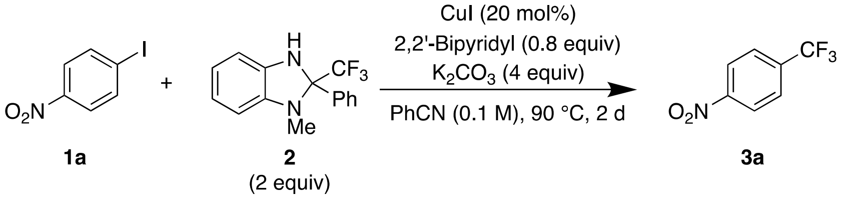

Aryl iodide **1** (0.1 mmol), trifluoromethylbenzimidazolidine **2** (56 mg, 0.2 mmol), CuI (3.8 mg, 0.02 mmol), 2,2'-bipyridine (12.5 mg, 0.08 mmol), and potassium carbonate (55.6 mg, 0.4 mmol) were mixed in benzonitrile (1.0 mL), and the mixture warmed at 90 °C. After 48 h, hexafluorobenzene was added as an internal standard and a ^{19}F NMR spectrum recorded for the calculation of the NMR yield. Then the crude products were purified by preparative TLC to give the trifluoromethylated products **3**.

Other hydroalkylation reactions in Figure 1 were performed based on this Procedure I.

Data of products

4-(Trifluoromethyl)nitrobenzene (3a)[S5)]

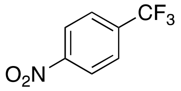

3a

^{1}H NMR (400 MHz, $CDCl_3$) δ 7.84 (d, *J* = 8.8 Hz, 2H), 8.36 (d, *J* = 8.8 Hz, 2H) ppm.

^{13}C NMR (100 MHz, $CDCl_3$) δ 122.9 (q, *J* = 272 Hz), 124.1, 126.8 (q, *J* = 3.6 Hz), 136.1 (q, *J* = 33 Hz), 150.0 ppm.

^{19}F NMR (376 MHz, $CDCl_3$, internal standard: $PhCF_3$; −63.7 ppm) δ −63.3 (s, 3F) ppm.

2-(Trifluoromethyl)nitrobenzene (3b)[S5)]

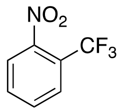

3b

^{1}H NMR (400 MHz, $CDCl_3$) δ 7.71–7.77 (m, 2H), 7.82–7.93 (m, 2H) ppm.

The pure product was difficult to provide sufficient amount for measurement of ^{13}C NMR due to its volatility. ^{13}C

NMR was measured from the mixture. ^{13}C NMR (100 MHz, $CDCl_3$) δ 121.9 (q, *J* = 272 Hz), 123.8 (q, *J* = 33.5 Hz), 125.0, 127.9 (q, *J* = 5.8 Hz), 132.5, 133.1, 148.2 ppm.

^{19}F NMR (376 MHz, $CDCl_3$, external standard: C_6F_6; −163.9 ppm) δ −58.4 (s, 3F) ppm.

3-(Trifluoromethyl)nitrobenzene (3c)[S5)]

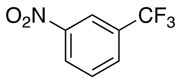

3c

^1H NMR (400 MHz, $CDCl_3$) δ 7.74 (d, *J* = 8.0 Hz, 1H), 7.98 (d, *J* = 8.0 Hz, 1H), 8.44 (d, *J* = 8.0 Hz, 1H), 8.51 (s, 1H) ppm.

^{13}C NMR (100 MHz, $CDCl_3$) δ 120.8 (q, *J* = 3.9 Hz), 122.8 (q, *J* = 271 Hz), 126.7, 130.3, 131.1 (q, *J* = 3.5 Hz), 132.3 (q, *J* = 34 Hz), 148.3 ppm.

^{19}F NMR (376 MHz, $CDCl_3$, internal standard: C_6F_6; −163.9 ppm) δ −65.1 (s, 3F) ppm.

4-(Trifluoromethyl)benzonitrile (3d)[S5)]

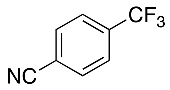

3d

^1H NMR (400 MHz, $CDCl_3$) δ 7.77 (d, *J* = 8.8 Hz, 2H), 7.82 (d, *J* = 8.8 Hz, 2H) ppm.

^{13}C NMR (100 MHz, $CDCl_3$) δ 116.0 (q, *J* = 1.8 Hz), 117.4, 123.0 (q, *J* = 271 Hz), 126.1 (q, *J* = 3.7 Hz), 132.6, 134.5 (q, *J* = 33 Hz) ppm.

^{19}F NMR (376 MHz, $CDCl_3$, external standard: C_6F_6; −163.9 ppm) δ −61.9 (s, 3F) ppm.

4'-Trifluoromethylacetophenone (3e)[S6)]

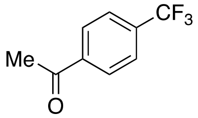

3e

^1H NMR (400 MHz, $CDCl_3$) δ 2.64 (s, 3H), 7.73 (d, *J* = 8.0 Hz, 2H), 8.06 (d, *J* = 8.0 Hz, 2H) ppm.

^{13}C NMR (100 MHz, $CDCl_3$) δ 26.8, 123.6 (q, *J* = 271 Hz), 125.7 (q, *J* = 3.7 Hz), 128.6, 134.4 (q, *J* = 32 Hz), 139.6, 197.0 ppm.

^{19}F NMR (376 MHz, $CDCl_3$, internal standard: $PhCF_3$; −63.7 ppm) δ −63.3 (s, 3F) ppm.

Methyl 4-trifluoromethylbenzoate (3f)[S7)]

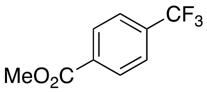

3f

^{1}H NMR (400 MHz, $CDCl_3$) δ 3.96 (s, 3H), 7.71 (d, *J* = 8.0 Hz, 2H), 8.16 (d, *J* = 7.6 Hz, 2H) ppm.

^{13}C NMR (100 MHz, $CDCl_3$) δ 52.5, 123.6 (q, *J* = 271 Hz), 125.4 (q, *J* = 4.0 Hz), 130.0, 133.3 (q, *J* = 1.5 Hz), 134.4 (q, *J* = 33 Hz), 165.9 ppm.

^{19}F NMR (376 MHz, $CDCl_3$, internal standard: $PhCF_3$; −63.7 ppm) δ −64.1 (s, 3F) ppm.

4-(Trifluoromethyl)benzaldehyde (3g)[S6)]

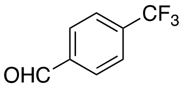

3g

^{1}H NMR (400 MHz, $CDCl_3$) δ 7.82 (d, *J* = 8.0 Hz, 2H), 8.02 (d, *J* = 8.0 Hz, 2H), 10.1 (s, 1H) ppm.

^{13}C NMR (100 MHz, $CDCl_3$) δ 123.4 (q, *J* = 271 Hz), 126.1 (q, *J* = 4.0 Hz), 129.9, 135.6 (q, *J* = 33 Hz), 138.6, 191.1 ppm.

^{19}F NMR (376 MHz, $CDCl_3$, external standard: C_6F_6; −163.9 ppm) δ −61.9 (s, 3F) ppm.

2-(Trifluoromethyl)anisole (3h)[S6)]

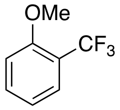

3h

The product could not be purified due to its volatility. The product was detected by ^{19}F NMR spectroscopy of the crude mixture.

^{19}F NMR (376 MHz, $CDCl_3$, external standard: C_6F_6; −163.9 ppm) δ −61.9 (s, 3F).

2-(Trifluoromethyl)pyridine (6a)[S5)]

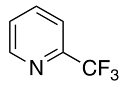

6a

The product could not be purified due to its volatility. ^{1}H NMR (mixture of some solvents, 400 MHz, $CDCl_3$) δ 7.51 (t, *J* = 7.6 Hz, 1H), 7.70 (d, *J* = 7.6 Hz, 1H), 7.89 (t, *J* = 7.6 Hz, 1H), 8.76 (d, *J* = 4.8 Hz, 1H) ppm.

^{19}F NMR (376 MHz, $CDCl_3$, external standard: C_6F_6; −163.9 ppm) δ −66.5 (s, 3F) ppm.

2-(Trifluoromethyl)quinoline (6b)[S8)]

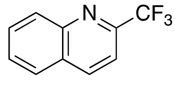

6b

^{1}H NMR (400 MHz, $CDCl_3$) δ 7.69 (td, *J* = 8.2, 1.2 Hz, 1H), 7.75 (d, *J* = 8.8 Hz, 1H), 7.81–7.86 (m, 1H), 7.92 (d, *J* = 8.0 Hz, 1H), 8.24 (d, *J* = 8.4 Hz, 1H), 8.37 (d, *J* = 8.8 Hz, 1H) ppm.

^{13}C NMR (100 MHz, $CDCl_3$) δ 116.8, 121.5 (q, *J* = 273 Hz), 127.7, 128.6, 128.3, 130.1, 130.8, 138.1, 147.1, 147.9 (q, *J* = 34.5 Hz) ppm.

^{19}F NMR (376 MHz, $CDCl_3$, external standard: C_6F_6; −163.9 ppm) δ −65.9 (s, 3F) ppm.

1-(Trifluoromethyl)isoquinoline (6c)[S9)]

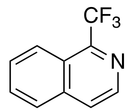

6c

^{1}H NMR (400 MHz, $CDCl_3$) δ 7.71–7.82 (m, 2H), 7.87 (d, *J* = 6.0 Hz, 1H), 7.94 (d, *J* = 7.6 Hz, 1H), 8.30–8.34 (m, 1H), 8.60 (d, *J* = 5.2 Hz, 1H) ppm.

^{13}C NMR (100 MHz, $CDCl_3$) δ 122.2 (q, *J* = 275 Hz), 124.5, 124.6, 124.7, 127.5, 128.8, 130.9, 137.1, 140.8, 146.4 (q, *J* = 32.6 Hz) ppm.

^{19}F NMR (376 MHz, $CDCl_3$, external standard: C_6F_6; −163.9 ppm) δ −61.4 (s, 3F) ppm.

2-(Trifluoromethyl)pyrazine (6d)

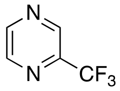

6d

The product could not be purified due to its volatility. The data were measured from the crude mixture, see: ^{1}H NMR (400 MHz, $CDCl_3$) δ 8.74 (m, 1H), 8.84 (m, 1H), 9.00 (s, 1H) ppm.

^{19}F NMR (376 MHz, $CDCl_3$, internal standard: $PhCF_3$; −63.7 ppm) δ −58.6 (s, 3F) ppm.

4. References

S1) M. Miyagawa, T. Ishikawa, K. Shinkai, T. Akiyama, *J. Fluorine Chem.* **2019**, *219*, 29–31.

S2) M. Kimber, P. I. Anderberg, M. M. Harding, *Tetrahedron* **2000**, *56*, 3575–3581.

S3) J. Clayden, S. P. Fletcher, J. J. W. McDouall, S. J. M. Rowbottom, *J. Am. Chem. Soc.* **2009**, *131*, 5331–5343.

S4) D. M. Wiemers, D. J. Burton, *J. Am. Chem. Soc.* **1986**, *108*, 832–834.

S5) H. Kondo, M. Oishi, K. Fujikawa, H. Amii, *Adv. Synth. Catal.* **2011**, *353,* 1247.

S6) H. Morimoto, T. Tsubogo, N. D. Litvinas, J. F. Hartwig, *Angew. Chem. Int. Ed.* **2011**, *50*, 3793.

S7) T. Knauber, F. Arikan, G.-V. Roschenthaler, L. J. Gooßen, *Chem. Eur. J.* **2011**, *17*, 2689.

S8) M. Oishi, H. Kondo, H. Amii, *Chem. Commun.* **2009**, *14*, 1909–1911.

S9) R. Pastor, A. Cambon, *J. Fluorine Chem.* **1979**, *13*, 279–296.

5. NMR spectra

S2b

^{1}H NMR (400 MHz, $CDCl_3$)

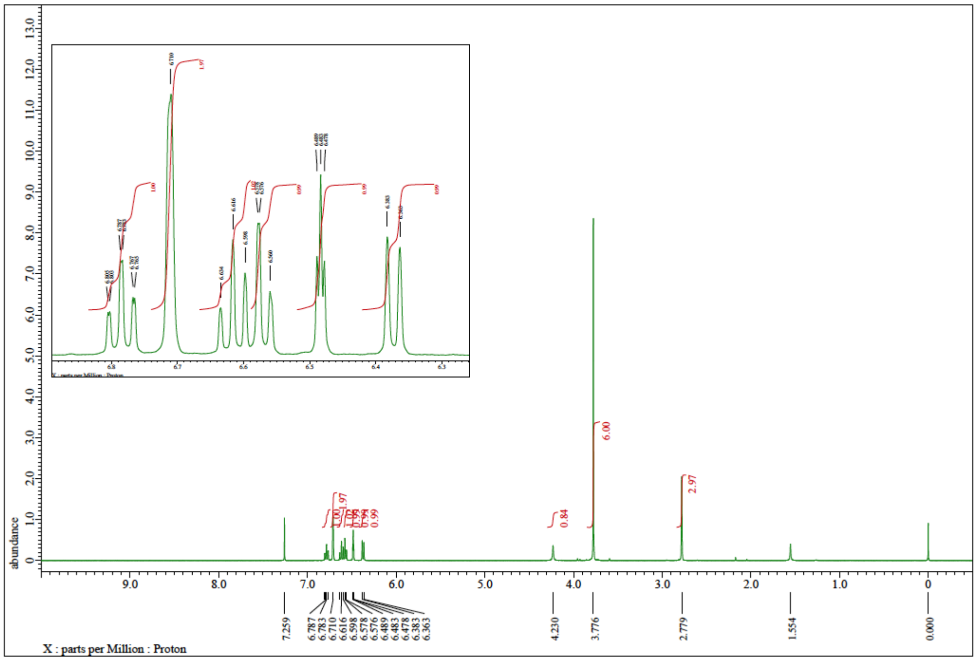

^{13}C NMR (100 MHz, $CDCl_3$)

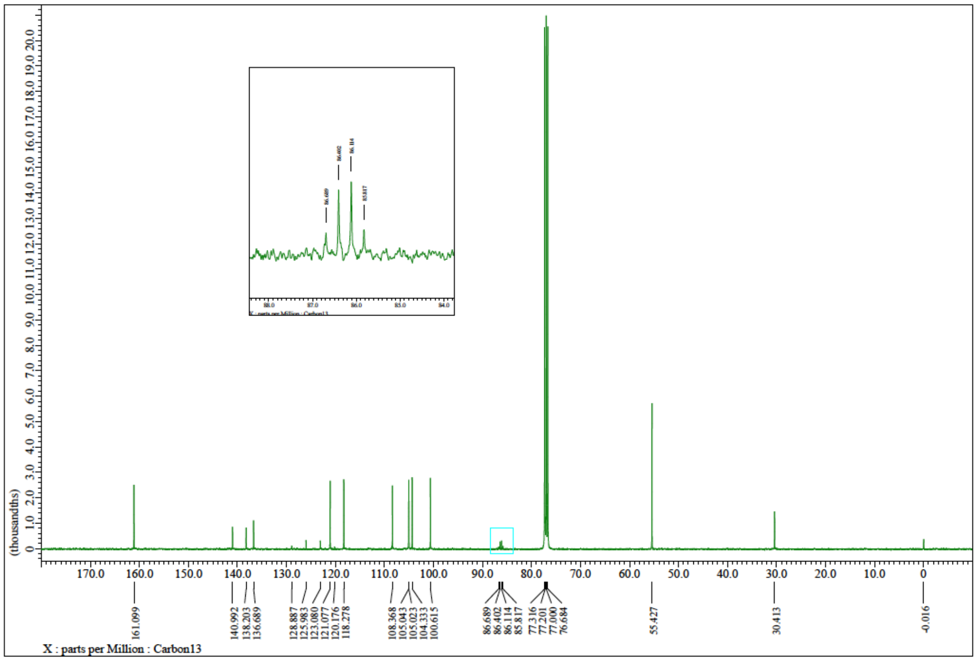

^{19}F NMR (376 MHz, $CDCl_3$)

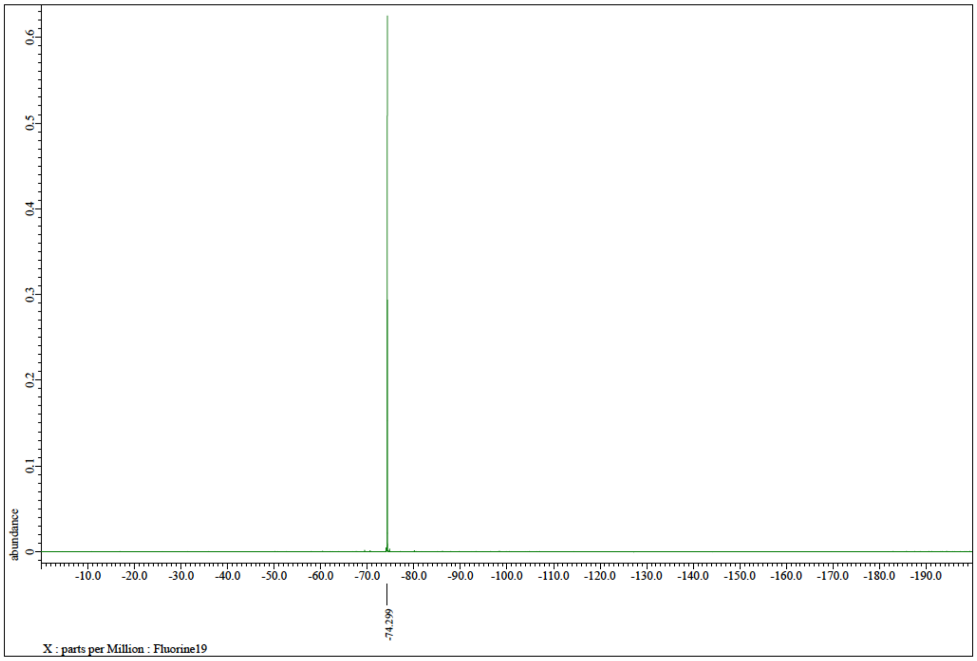

Hexafluorobenzene (−163.9 ppm) was used as external standard.

S2c

^{1}H NMR (400 MHz, $CDCl_3$)

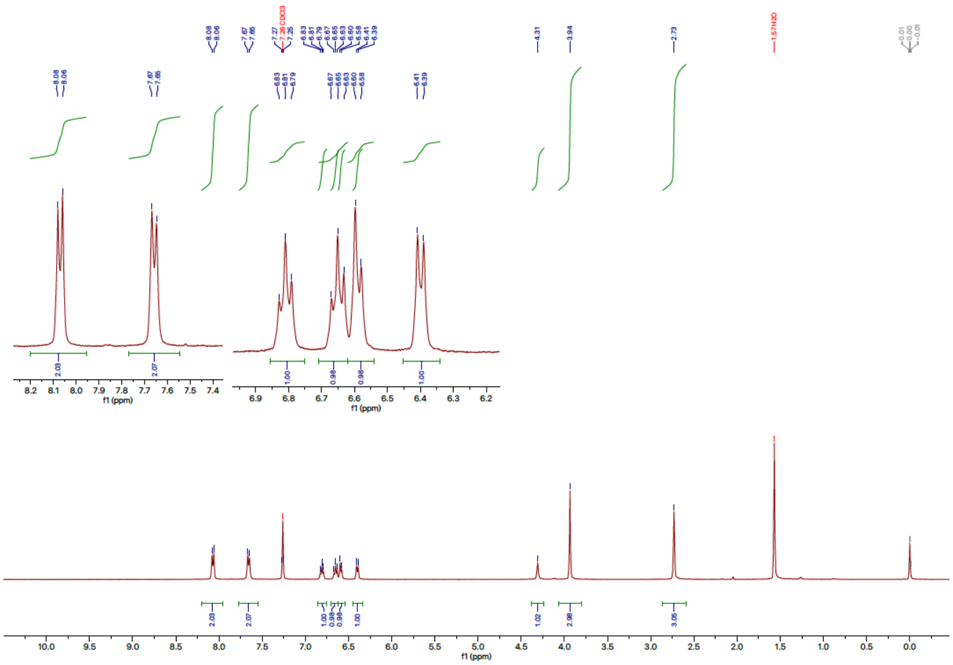

^{13}C NMR (100 MHz, $CDCl_3$)

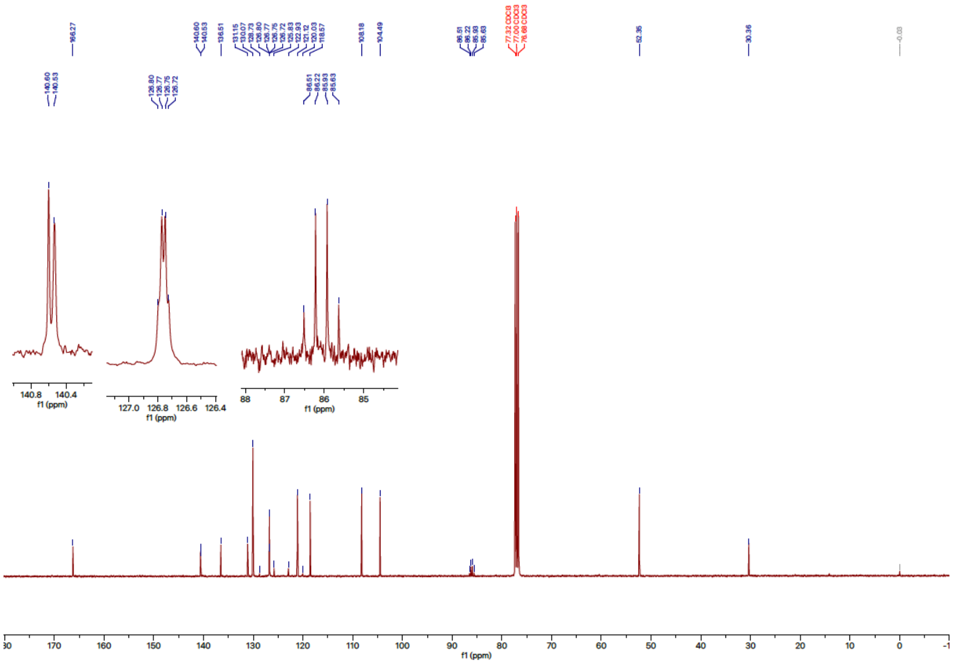

^{19}F NMR (376 MHz, $CDCl_3$)

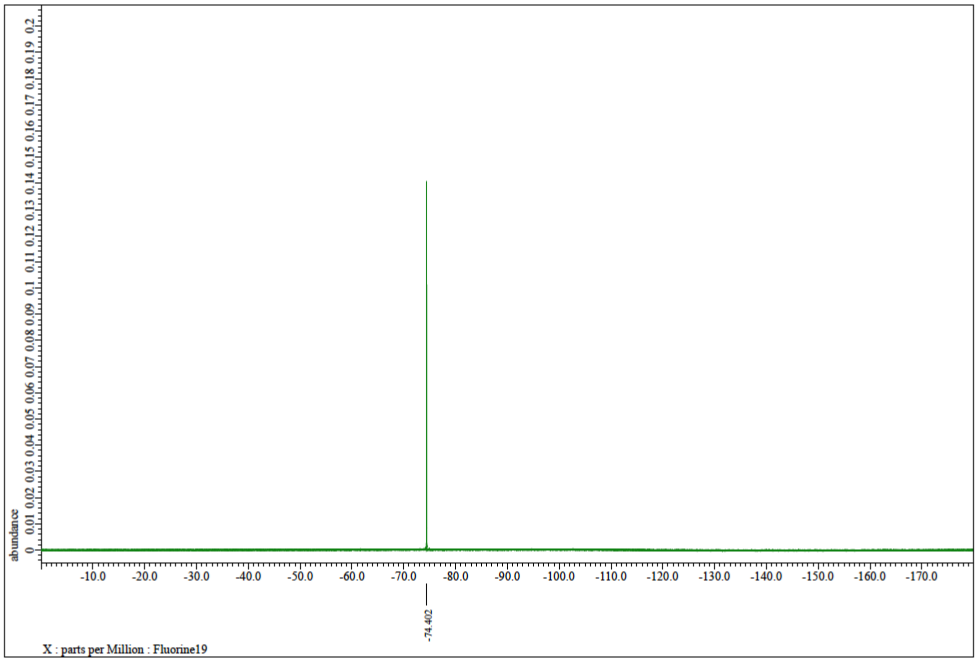

Hexafluorobenzene (−163.9 ppm) was used as external standard.

S2d

^{1}H NMR (400 MHz, CDCl$_3$)

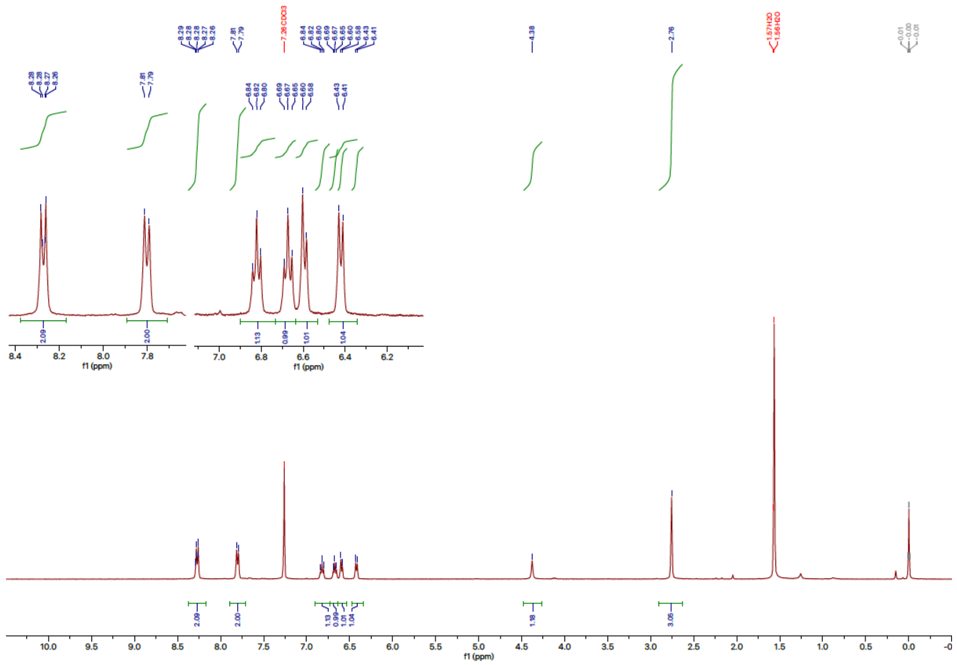

^{13}C NMR (100 MHz, CDCl$_3$)

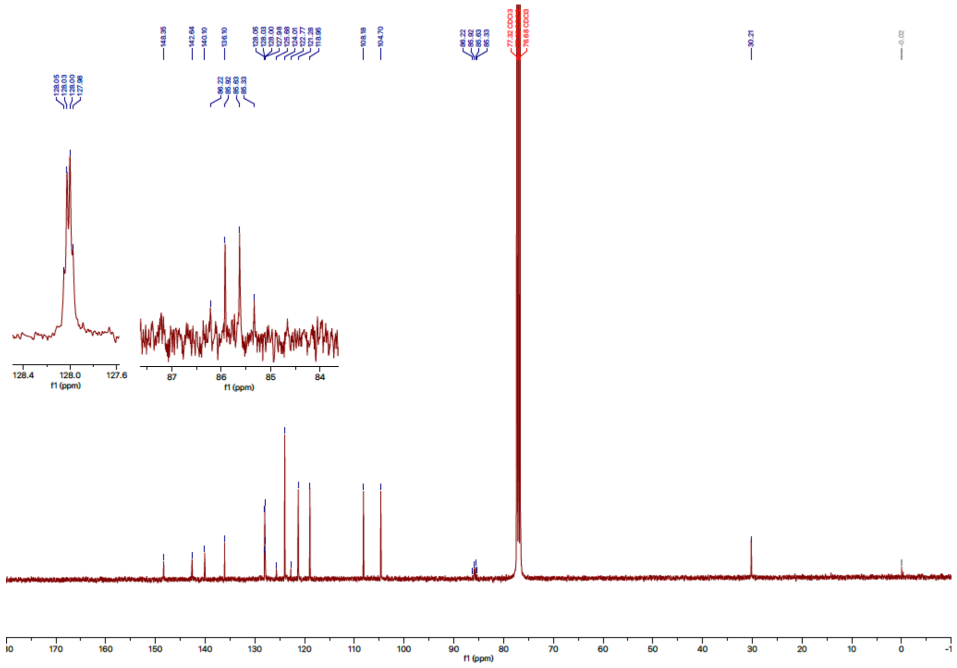

^{19}F NMR (376 MHz, CDCl$_3$)

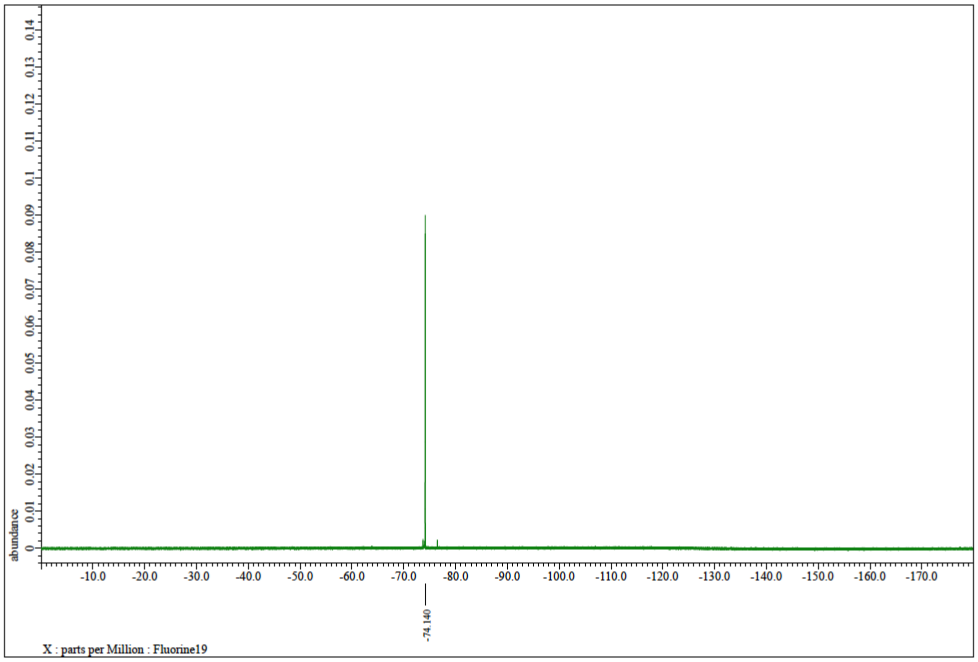

Hexafluorobenzene (− 163.9 ppm) was used as external standard.

3a

^{1}H NMR (400 MHz, $CDCl_3$)

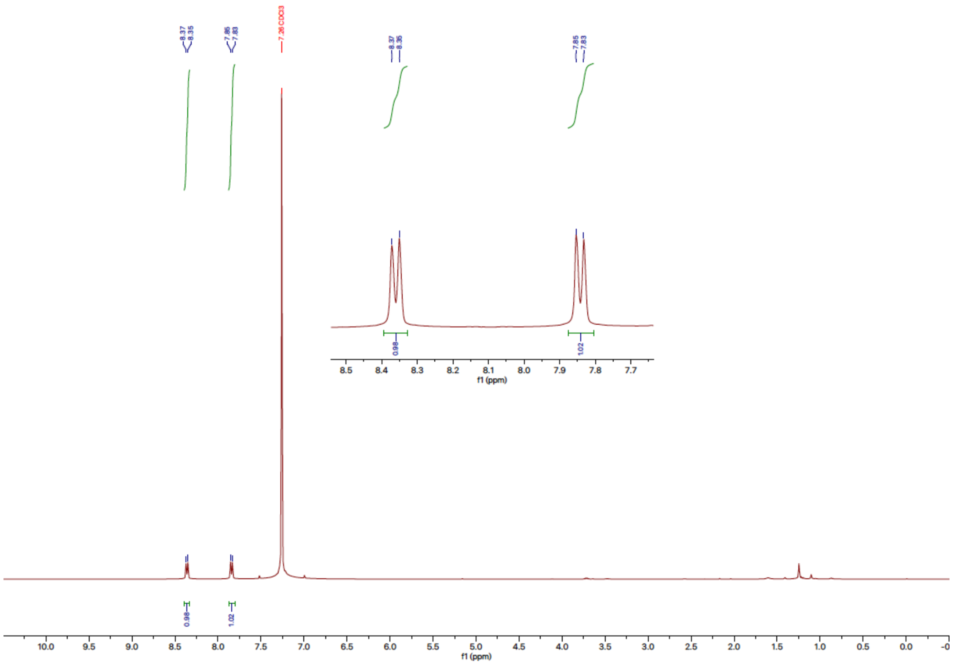

^{13}C NMR (100 MHz, $CDCl_3$)

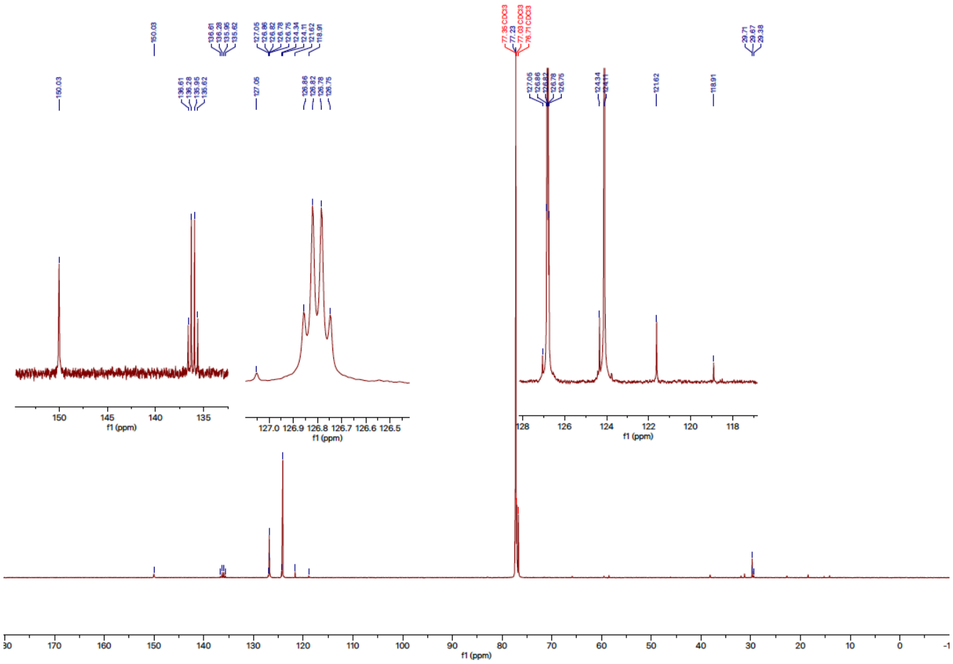

^{19}F NMR (376 MHz, $CDCl_3$)

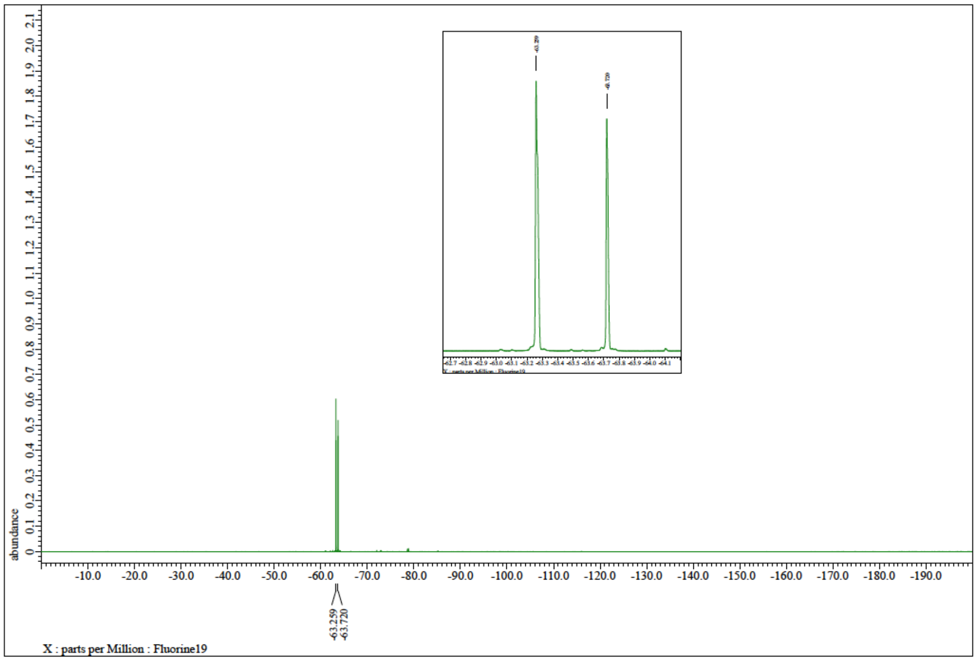

Benzotrifluoride (−63.7 ppm) was used as internal standard.

3b

^{1}H NMR (400 MHz, $CDCl_3$)

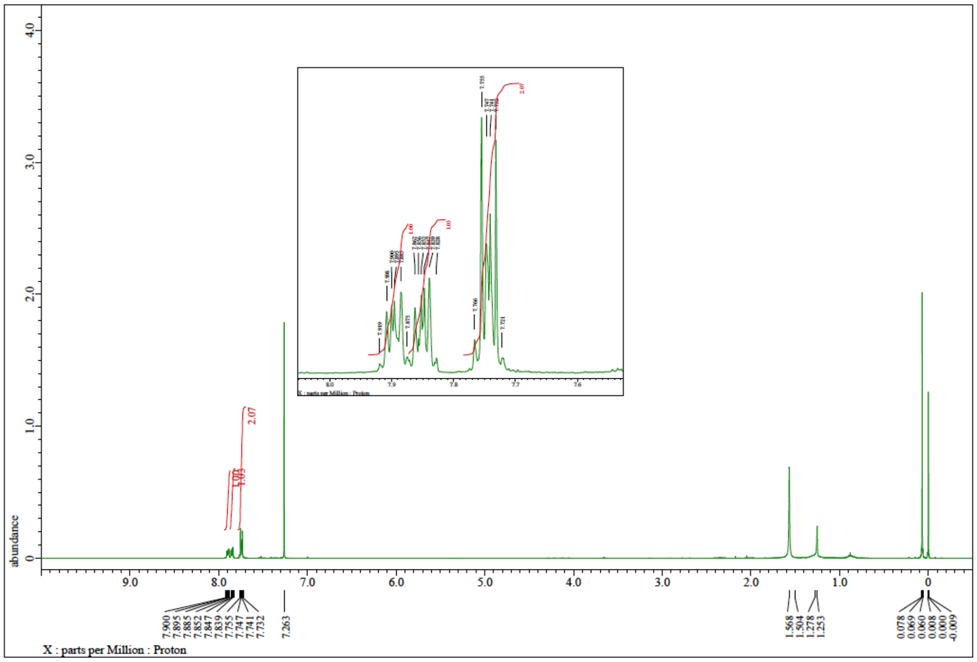

^{13}C NMR (100 MHz, $CDCl_3$)

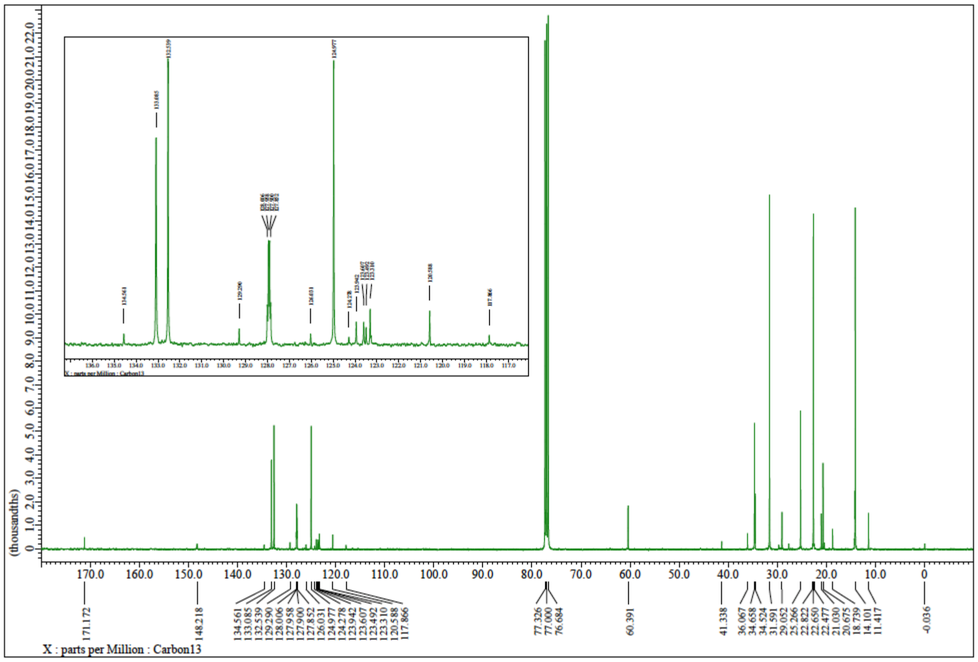

^{19}F NMR (376 MHz, $CDCl_3$)

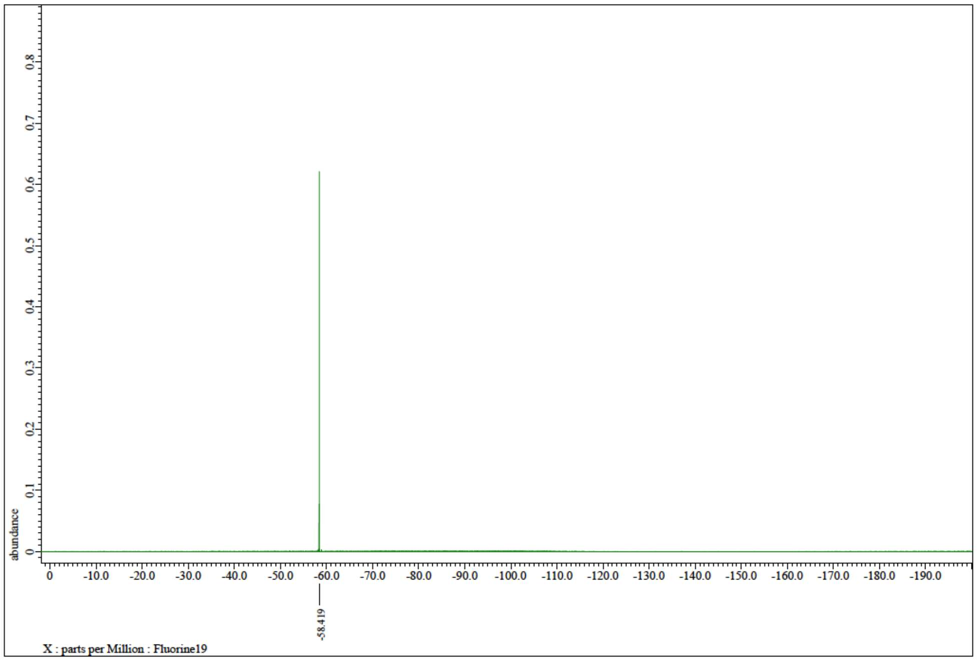

Hexafluorobenzene (− 163.9 ppm) was used as external standard.

3c

^{1}H NMR (400 MHz, $CDCl_3$)

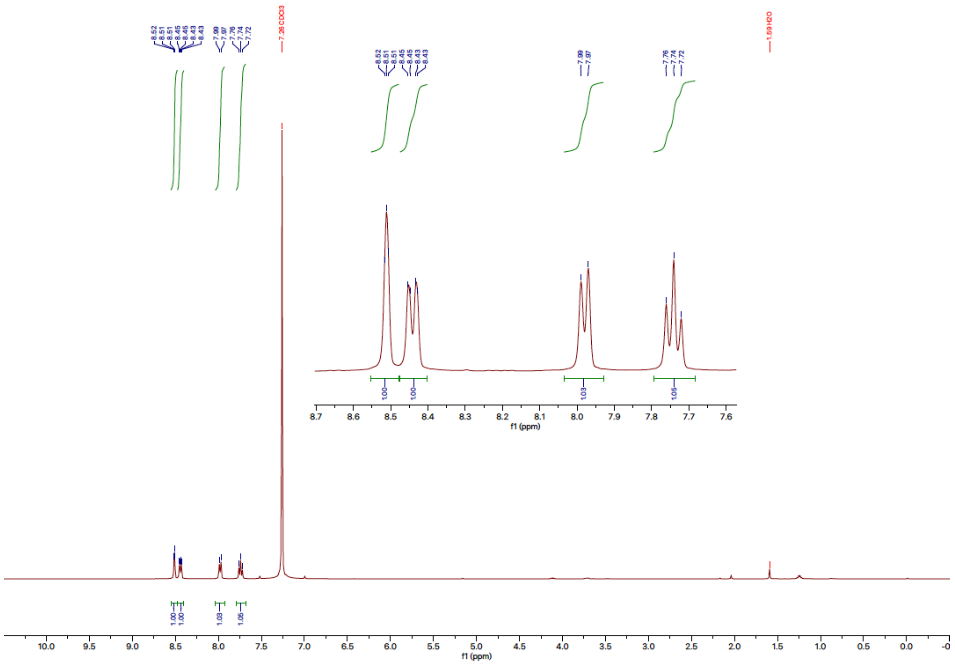

^{13}C NMR (100 MHz, $CDCl_3$)

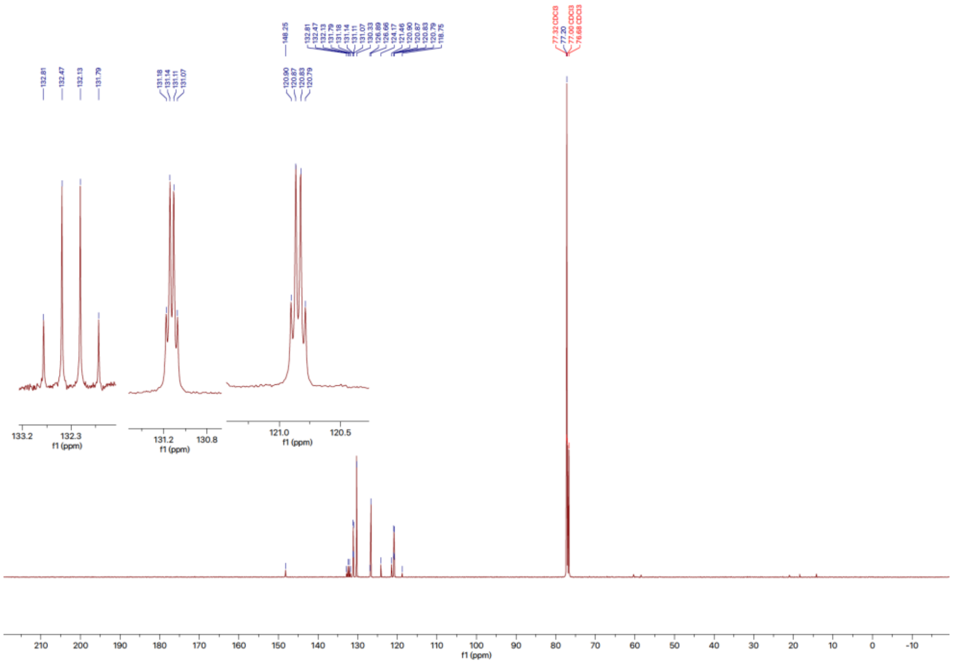

^{19}F NMR (376 MHz, CDCl$_3$)

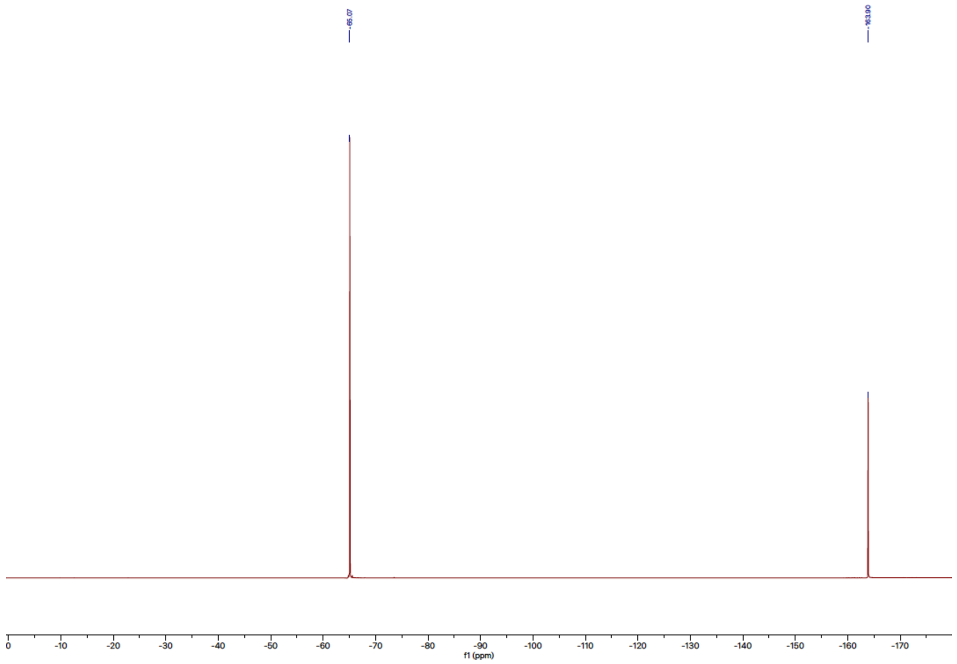

Hexafluorobenzene (−163.9 ppm) was used as internal standard.

3d

^{1}H NMR (400 MHz, CDCl$_3$)

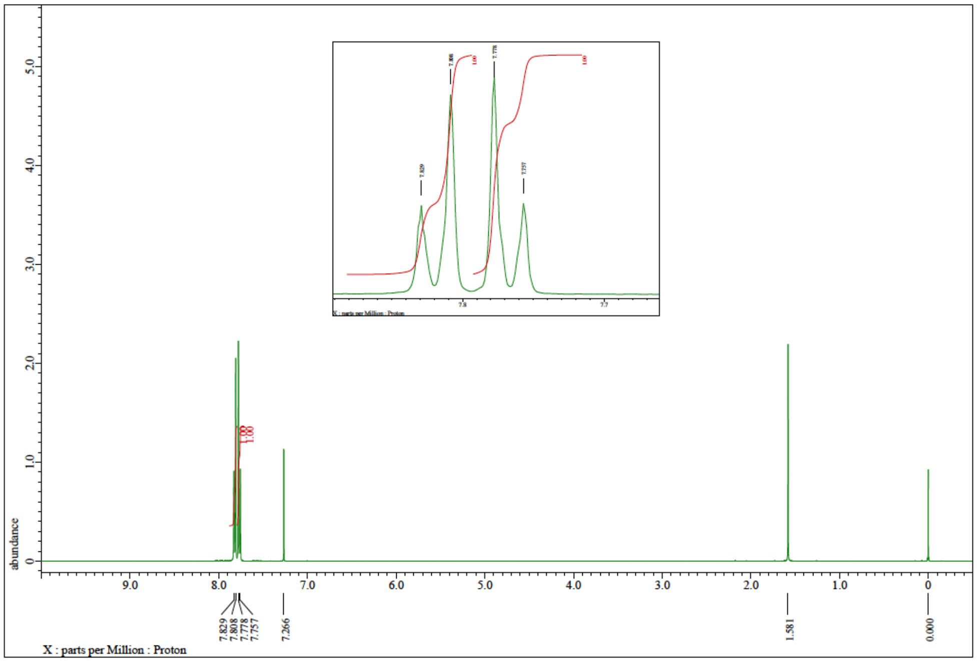

^{13}C NMR (100 MHz, CDCl$_3$)

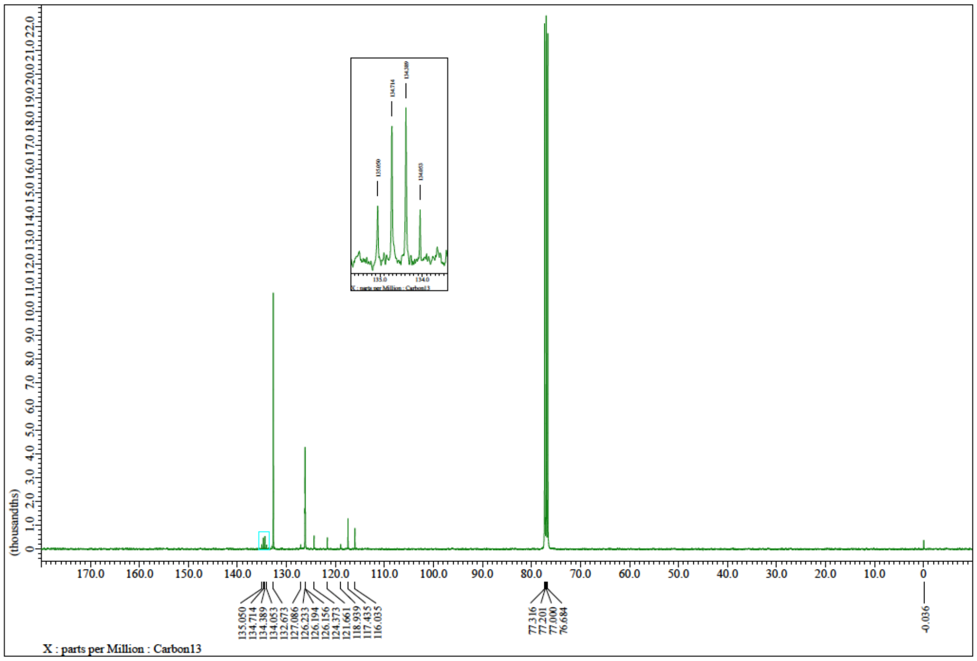

^{19}F NMR (376 MHz, $CDCl_3$)

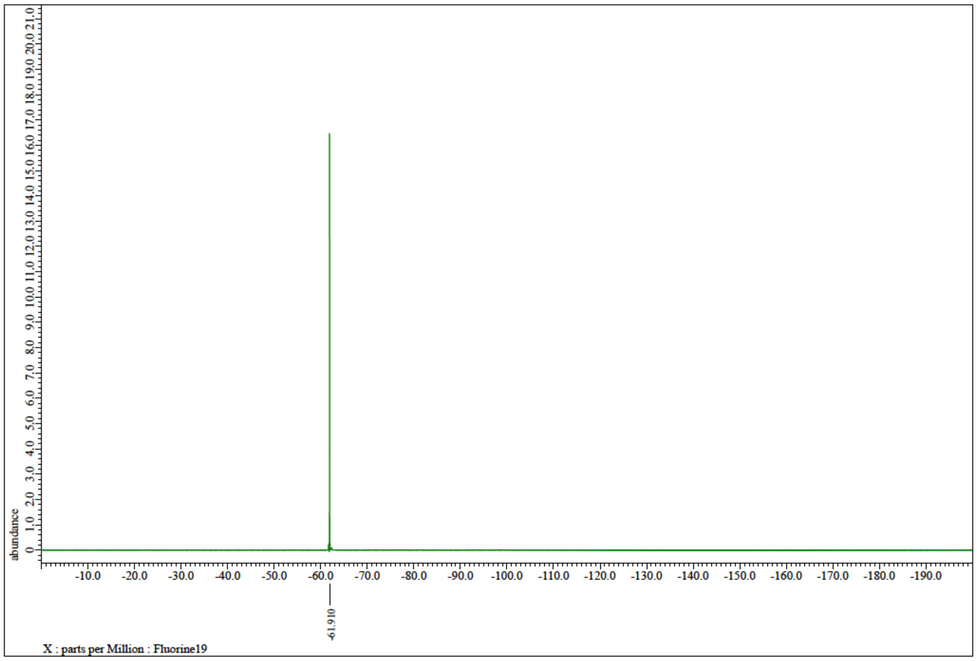

Hexafluorobenzene (−163.9 ppm) was used as external standard.

3e

^{1}H NMR (400 MHz, $CDCl_3$)

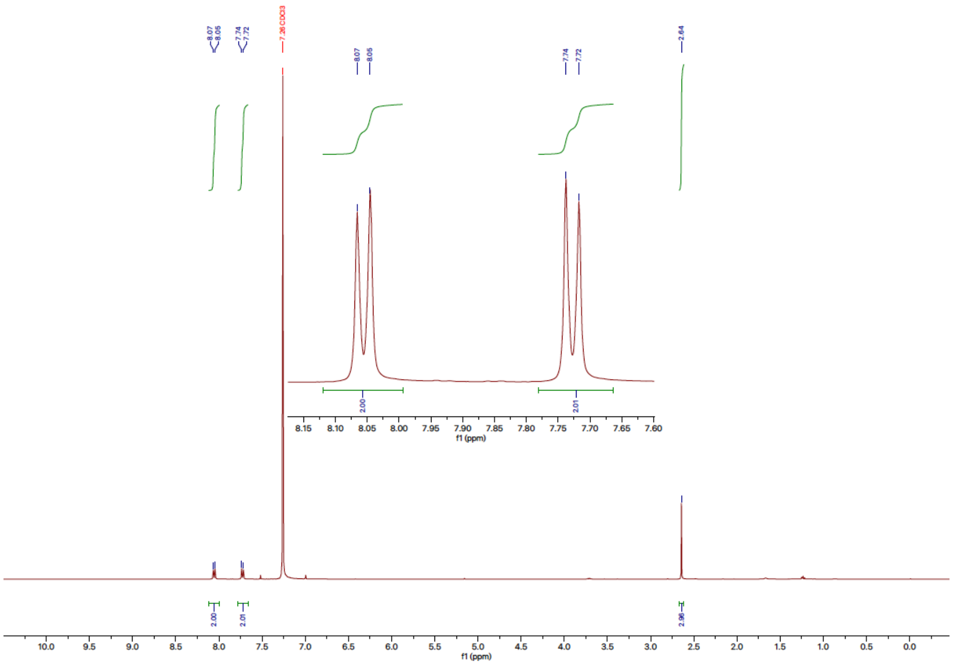

^{13}C NMR (100 MHz, $CDCl_3$)

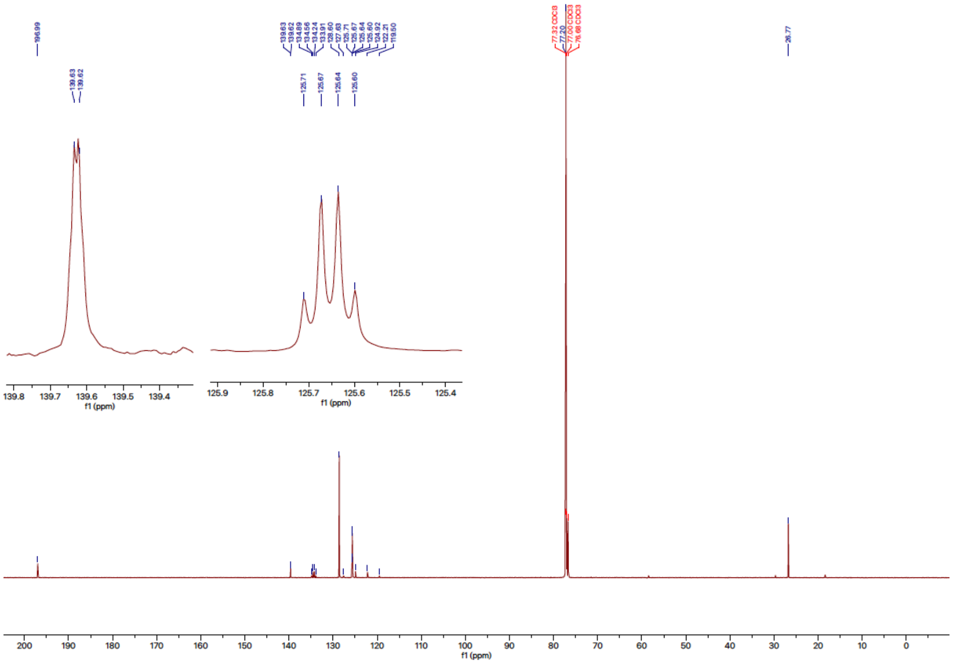

^{19}F NMR (376 MHz, $CDCl_3$)

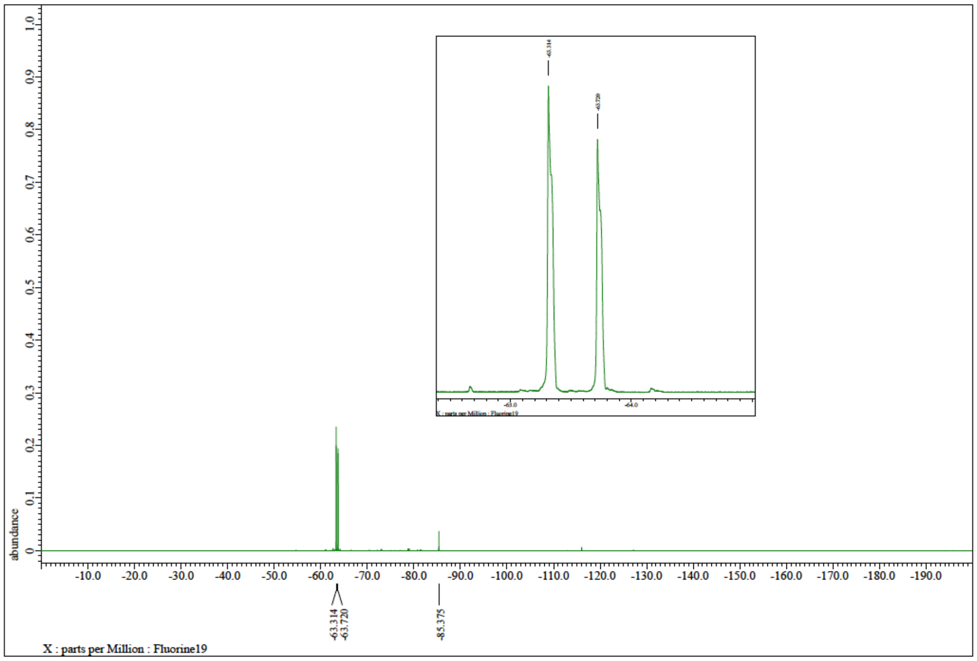

Benzotrifluoride (−63.7 ppm) was used as internal standard.

3f

^{1}H NMR (400 MHz, $CDCl_3$)

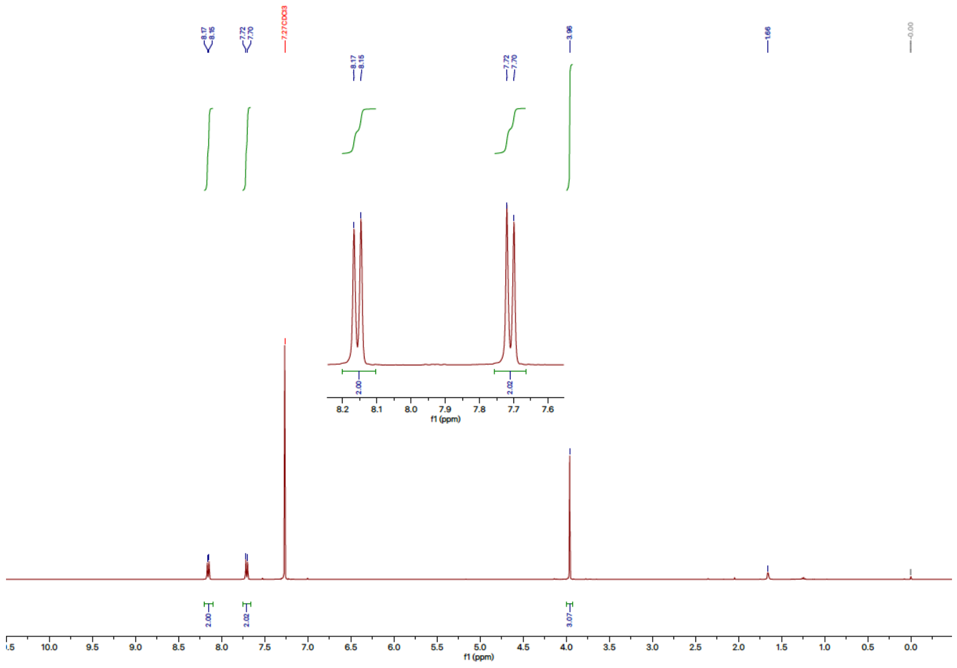

^{13}C NMR (100 MHz, $CDCl_3$)

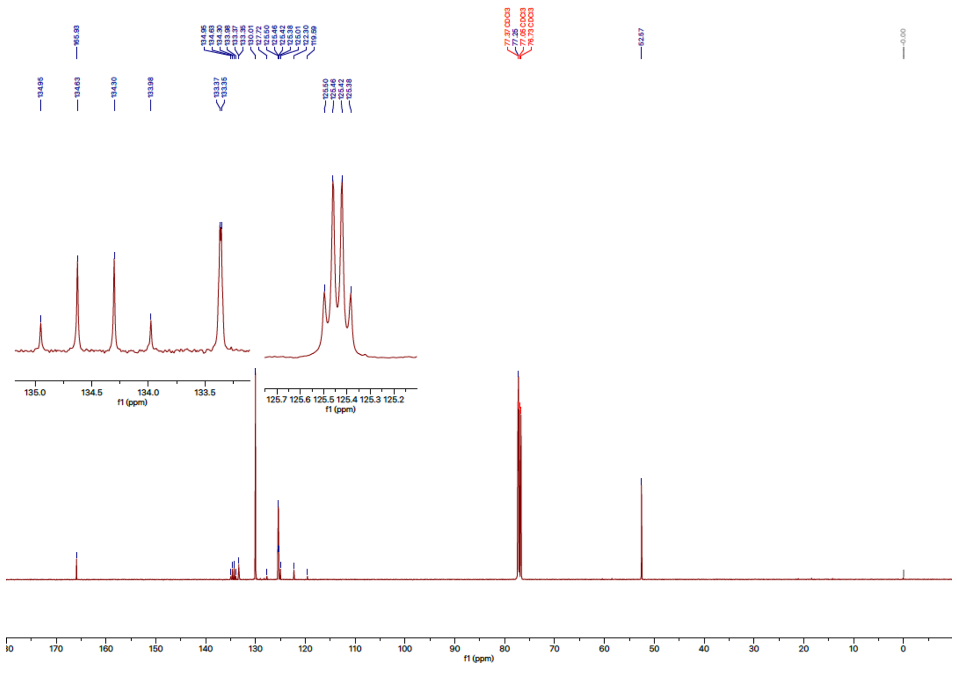

^{19}F NMR (376 MHz, $CDCl_3$)

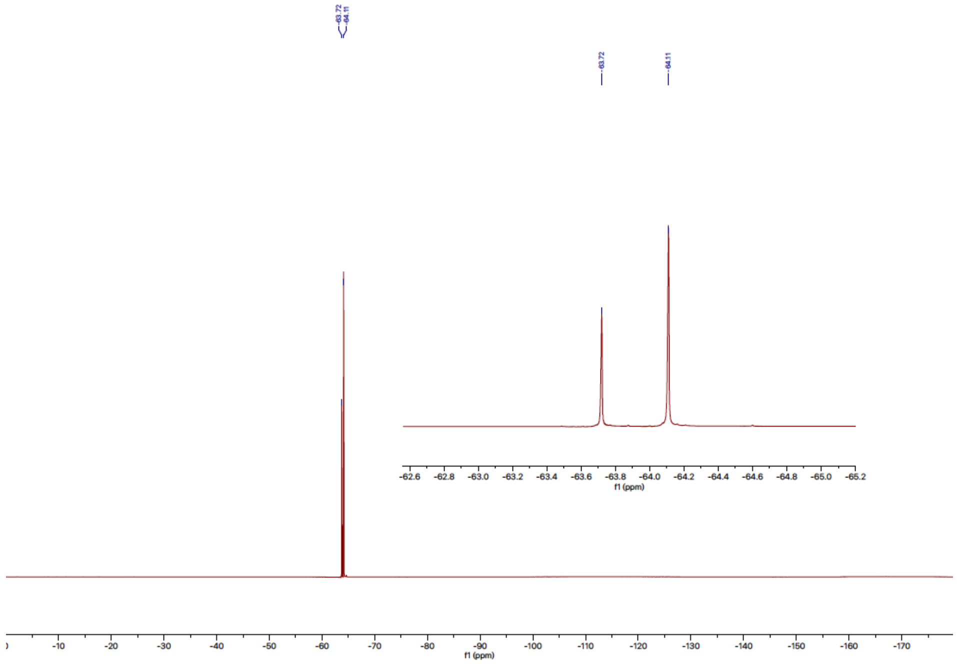

Benzotrifluoride (−63.7 ppm) was used as internal standard.

3g

^{1}H NMR (400 MHz, $CDCl_3$)

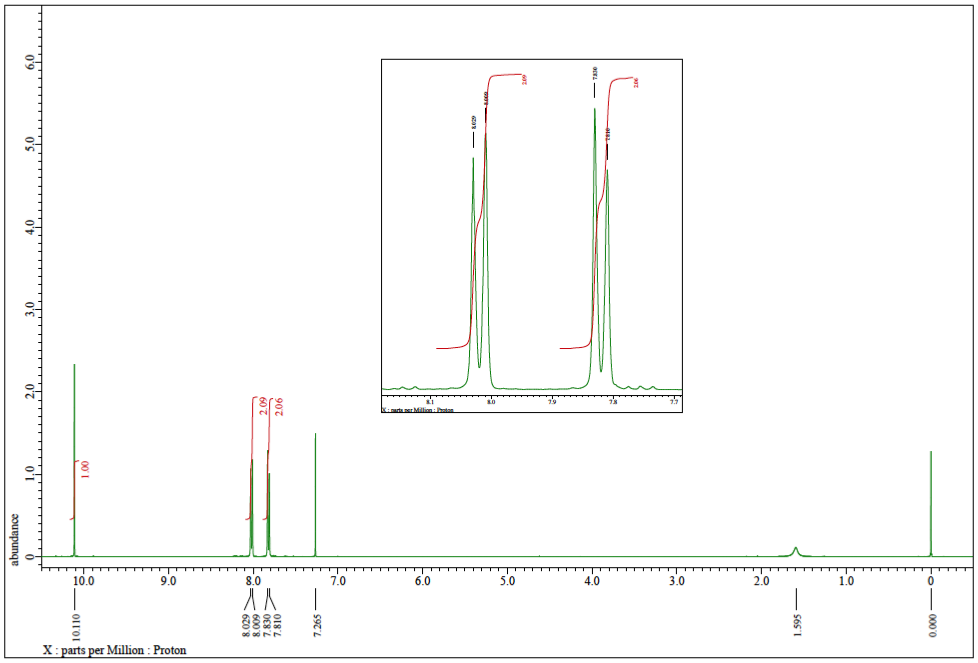

^{13}C NMR (100 MHz, $CDCl_3$)

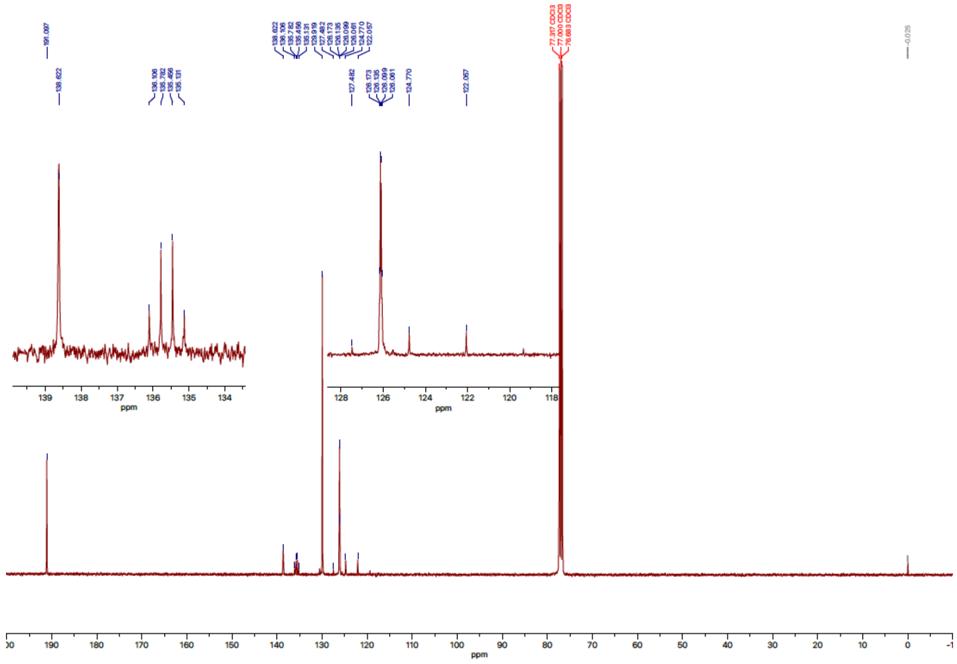

^{19}F NMR (376 MHz, CDCl$_3$)

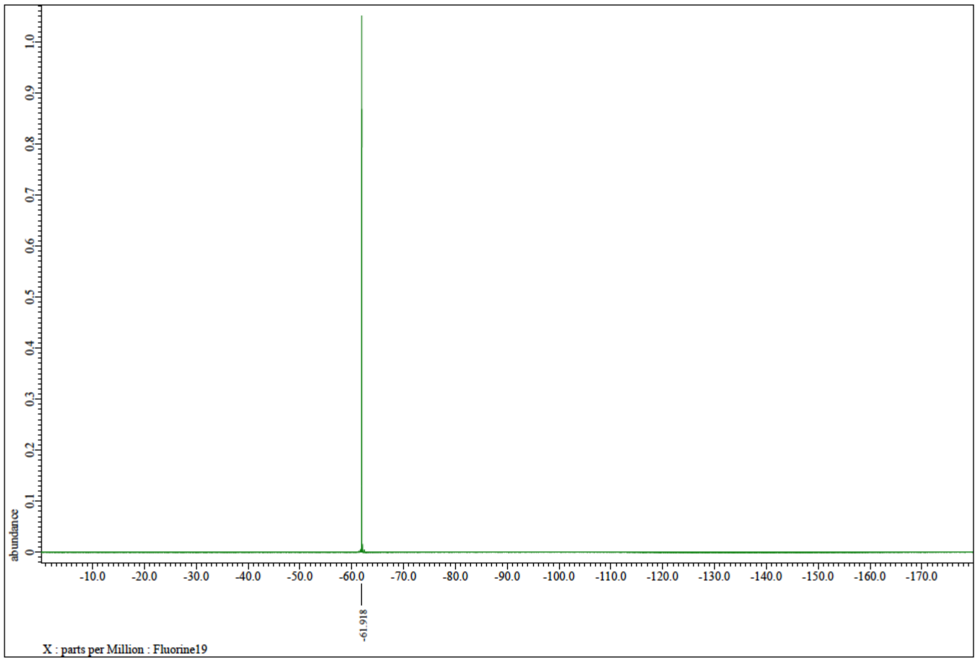

Hexafluorobenzene (− 163.9 ppm) was used as external standard.

3h

^{19}F NMR (Reaction mixture)

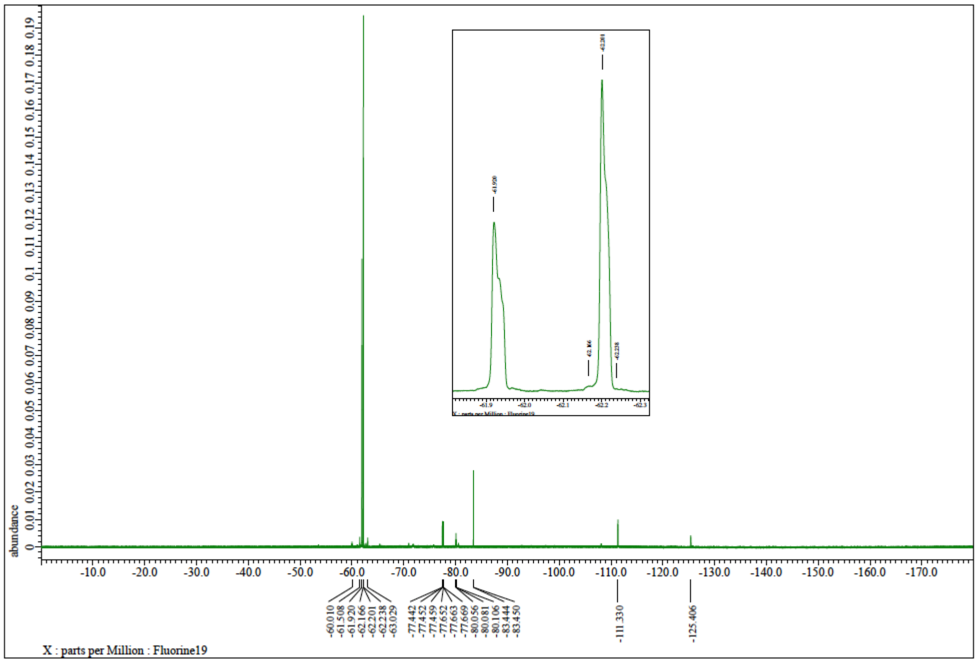

Hexafluorobenzene (− 163.9 ppm) was used as external standard.

6a

^{1}H NMR (400 MHz, $CDCl_3$)

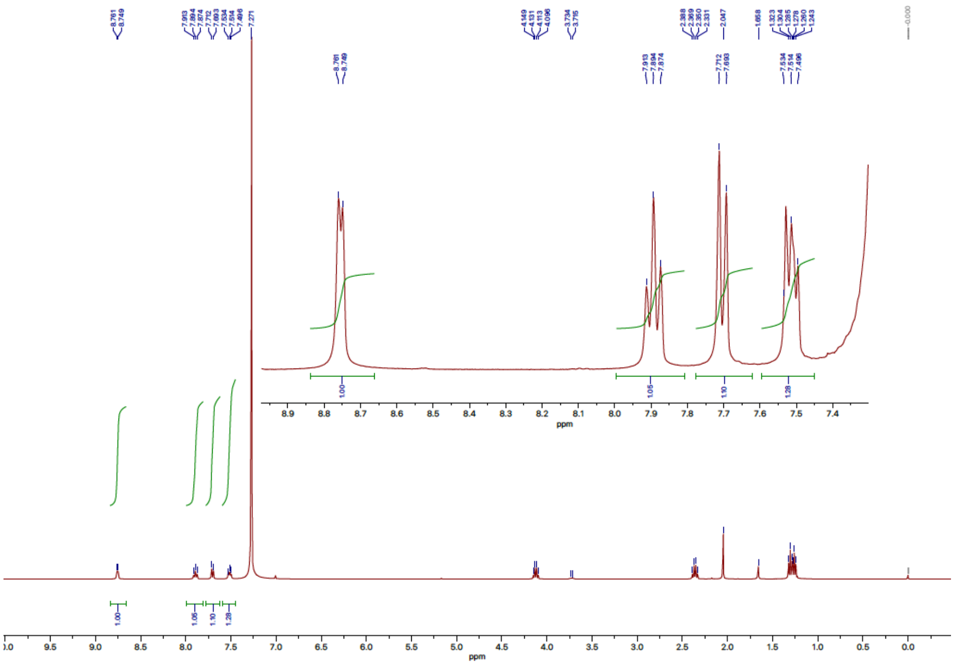

^{19}F NMR (376 MHz, $CDCl_3$)

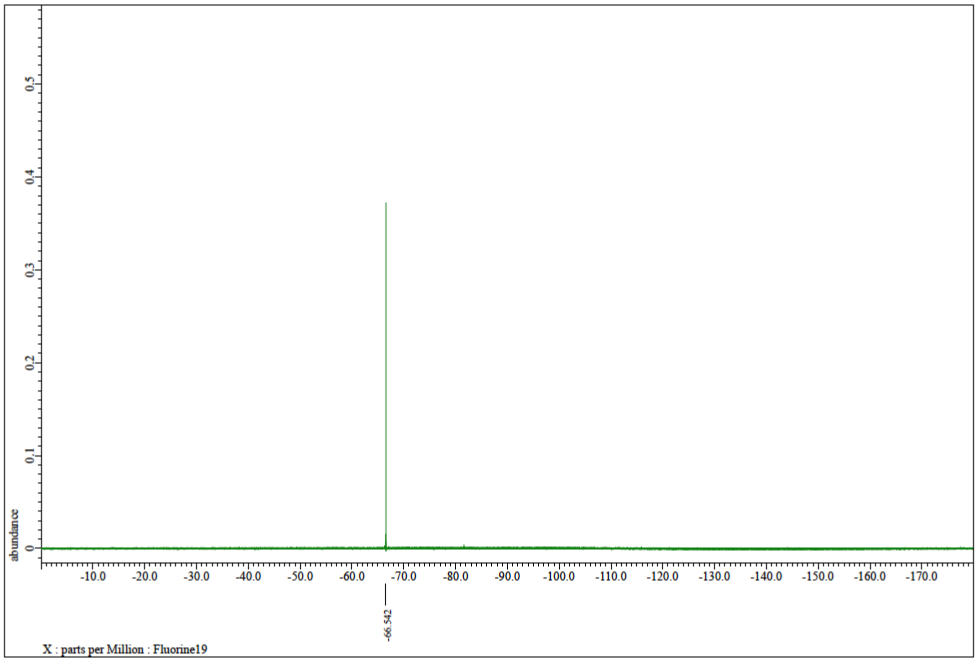

Hexafluorobenzene (−163.9 ppm) was used as external standard.

6b

^{1}H NMR (400 MHz, $CDCl_3$)

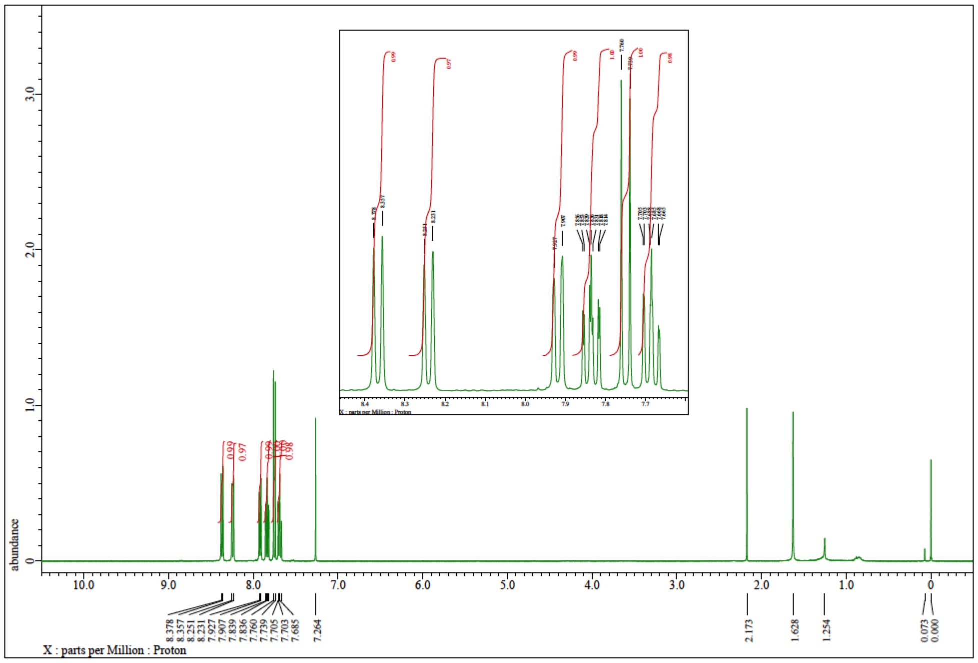

^{13}C NMR (100 MHz, $CDCl_3$)

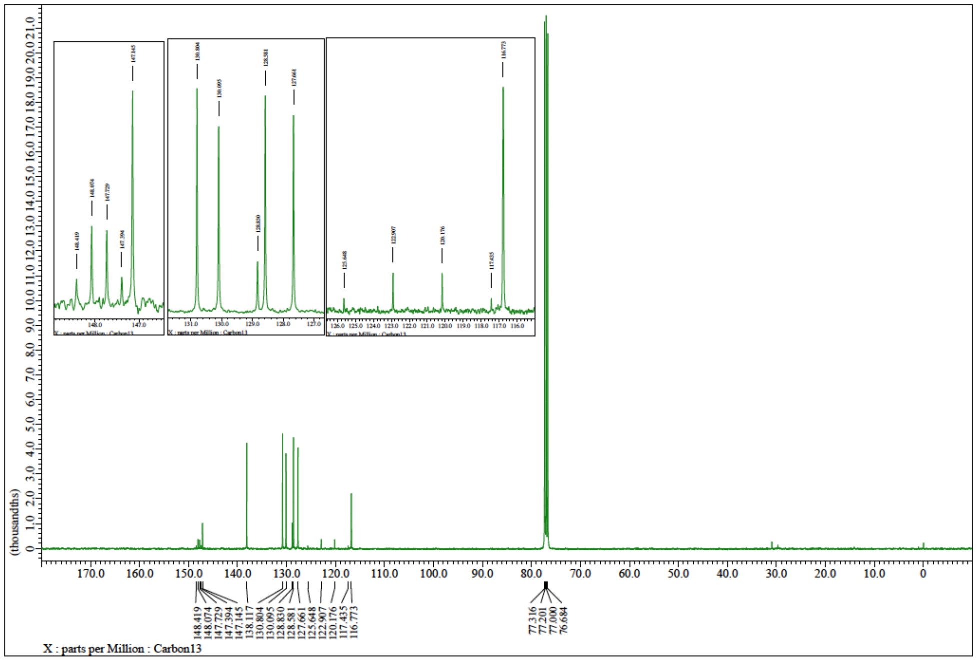

^{19}F NMR (376 MHz, $CDCl_3$)

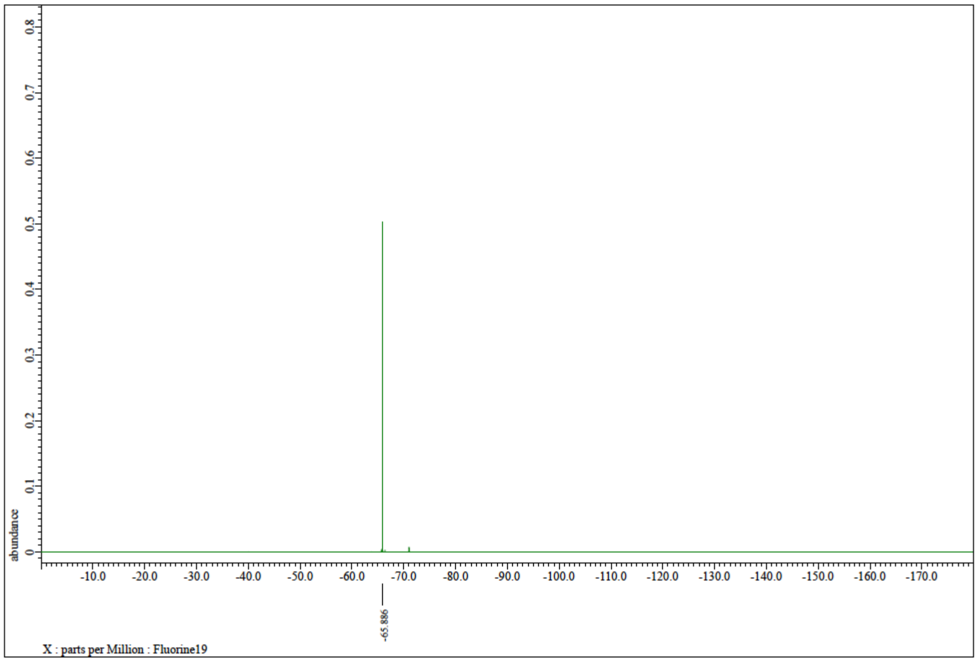

Hexafluorobenzene (−163.9 ppm) was used as external standard.

6c

^{1}H NMR (400 MHz, $CDCl_3$)

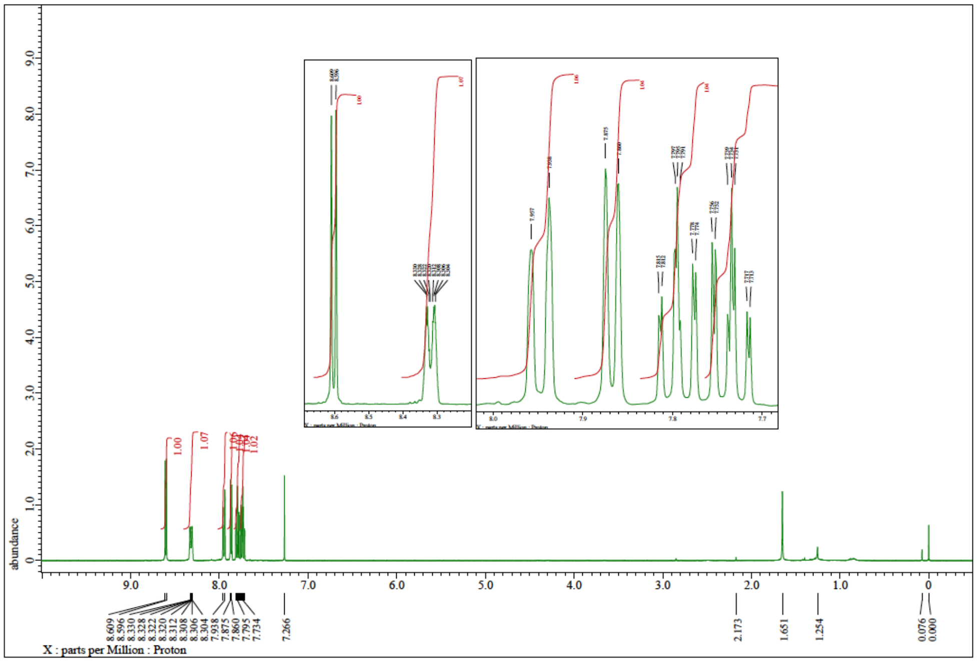

^{13}C NMR (100 MHz, $CDCl_3$)

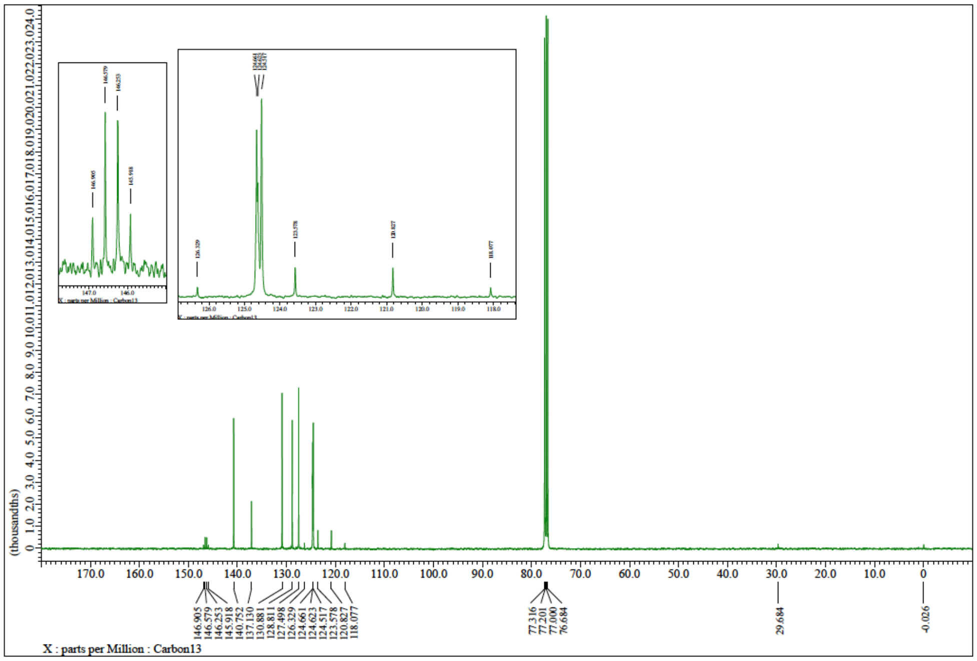

^{19}F NMR (376 MHz, $CDCl_3$)

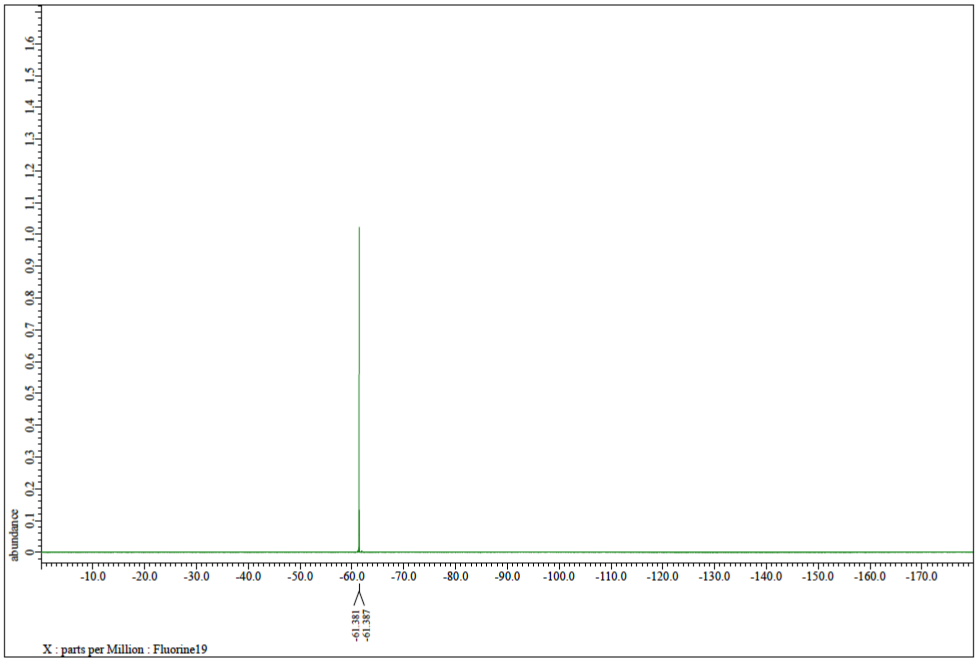

Hexafluorobenzene (− 163.9 ppm) was used as external standard.

6d

^{19}F NMR (376 MHz, $CDCl_3$)

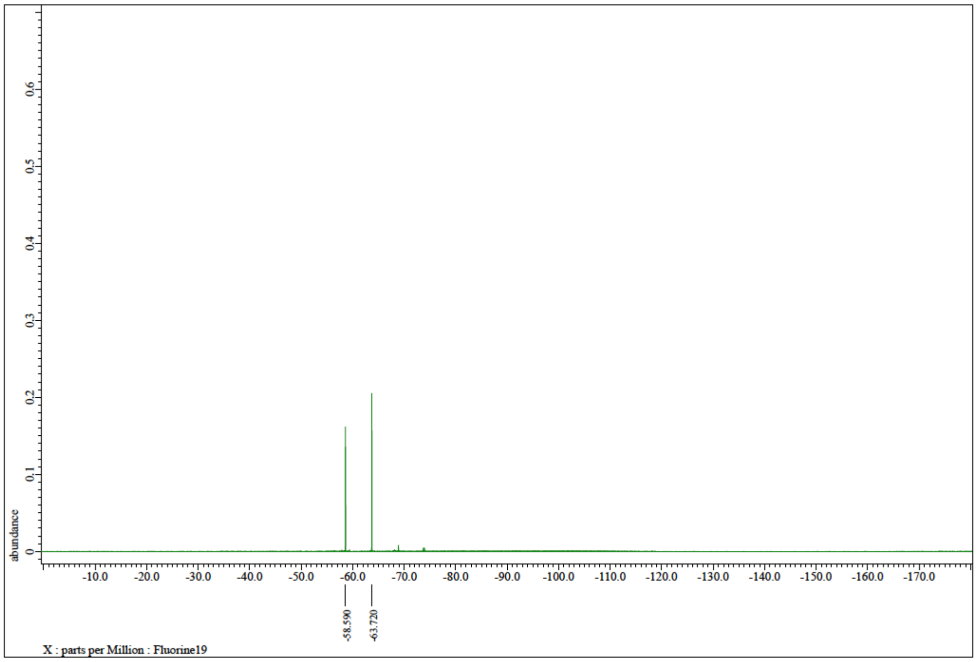

Benzotrifluoride (−63.7 ppm) was used as internal standard.
